# Landscape-Level Variation in Disease Susceptibility Related to Shallow-Water Hypoxia

**DOI:** 10.1371/journal.pone.0116223

**Published:** 2015-02-11

**Authors:** Denise L. Breitburg, Darryl Hondorp, Corinne Audemard, Ryan B. Carnegie, Rebecca B. Burrell, Mark Trice, Virginia Clark

**Affiliations:** 1 Smithsonian Environmental Research Center, PO Box 28, Edgewater, MD, 21037, United States of America; 2 USGS Great Lakes Science Center, 1451 Green Road, Ann Arbor, MI, 48105, United States of America; 3 Virginia Institute of Marine Science, College of William and Mary, PO Box 1346, Gloucester Point, VA, 23062, United States of America; 4 Maryland Department of Natural Resources, 580 Taylor Avenue, Annapolis, MD, 21401, United States of America; University of Hong Kong, HONG KONG

## Abstract

Diel-cycling hypoxia is widespread in shallow portions of estuaries and lagoons, especially in systems with high nutrient loads resulting from human activities. Far less is known about the effects of this form of hypoxia than deeper-water seasonal or persistent low dissolved oxygen. We examined field patterns of diel-cycling hypoxia and used field and laboratory experiments to test its effects on acquisition and progression of *Perkinsus marinus* infections in the eastern oyster, *Crassostrea virginica*, as well as on oyster growth and filtration. *P. marinus* infections cause the disease known as Dermo, have been responsible for declines in oyster populations, and have limited success of oyster restoration efforts. The severity of diel-cycling hypoxia varied among shallow monitored sites in Chesapeake Bay, and average daily minimum dissolved oxygen was positively correlated with average daily minimum pH. In both field and laboratory experiments, diel-cycling hypoxia increased acquisition and progression of infections, with stronger results found for younger (1-year-old) than older (2-3-year-old) oysters, and more pronounced effects on both infections and growth found in the field than in the laboratory. Filtration by oysters was reduced during brief periods of exposure to severe hypoxia. This should have reduced exposure to waterborne *P. marinus*, and contributed to the negative relationship found between hypoxia frequency and oyster growth. Negative effects of hypoxia on the host immune response is, therefore, the likely mechanism leading to elevated infections in oysters exposed to hypoxia relative to control treatments. Because there is considerable spatial variation in the frequency and severity of hypoxia, diel-cycling hypoxia may contribute to landscape-level spatial variation in disease dynamics within and among estuarine systems.

## Introduction

Hypoxia or low dissolved oxygen (DO) is a pervasive problem in estuaries and nearshore coastal ecosystems [1,2], and typically results from a combination of physical characteristics that limit re-aeration and anthropogenic nutrient enrichment that increases system production and oxygen consumption. Most research has focused on bottom-layer oxygen depletion that persists from days to millennia and can cause a wide range of effects including increased mortality, reduced growth, altered behavior, and changes in food webs and biogeochemical cycles [1,3,4]. Prolonged exposure to hypoxia has been shown to increase pathogen-related mortality and impair immune responses in a number of marine and estuarine fish and invertebrates [5–8].

**Fig 1:**
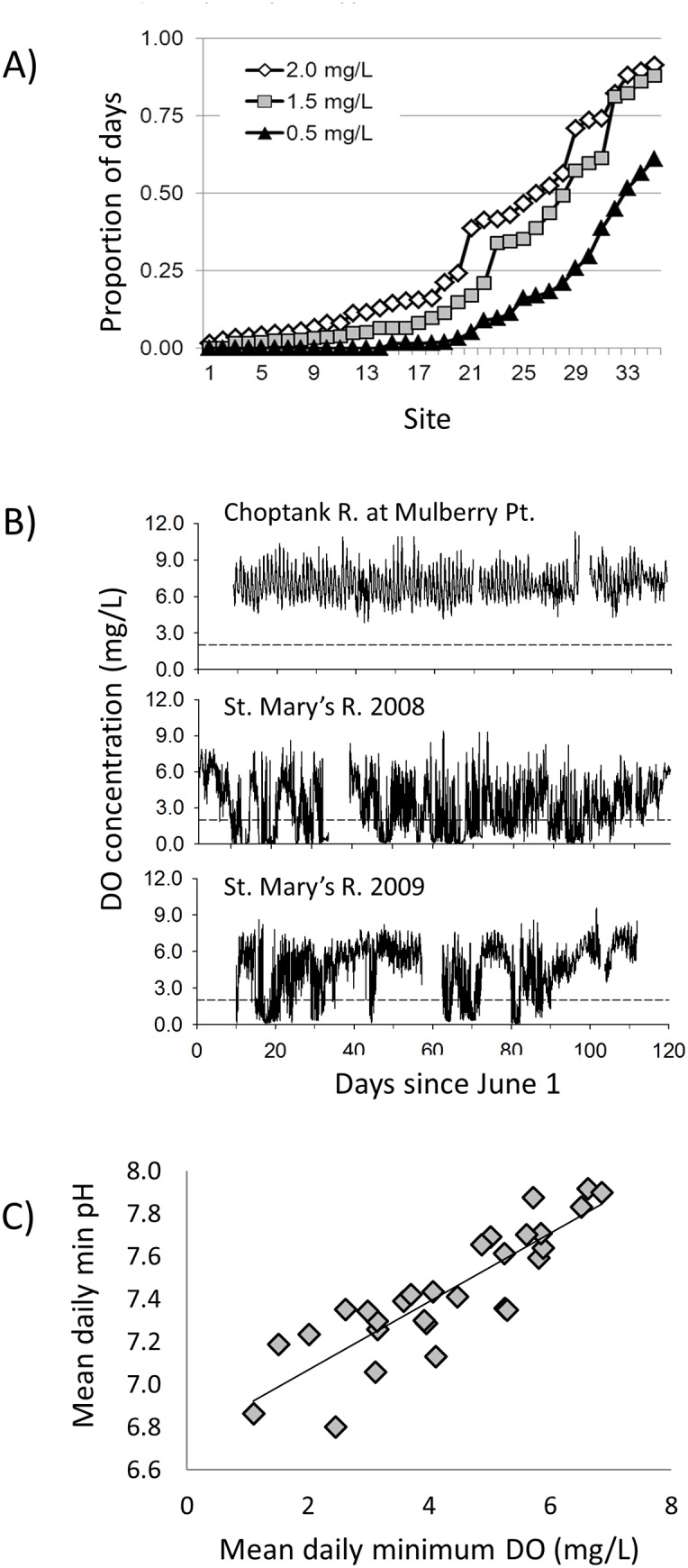
Patterns of diel-cycling dissolved oxygen concentrations in shallow (<2m) water in Chesapeake Bay and Maryland coastal bays. (A) Proportion of days during July and August with minimum DO concentrations ≤2.0, 1.5 and 0.5 mg l^-1^ at 36 sites with mean summer salinity ≤7.0 that were monitored ≤0.5 m above the bottom between 2001 and 2012. Note that site numbers do not correspond to field experiment sites and are used only to order the sites from least to most frequent hypoxia. (B) DO concentrations at two of the field deployment sites. DO concentrations at Mulberry Pt. always remained above 50% saturation. In contrast DO periodically dropped below 0.5 mg l^-1^ at the St. Mary’s R. site, and had a higher frequency of days with DO <0.5 mg l^-1^ in 2008 than in 2009. The reference line at 2.0 mg l^-1^ indicates conditions typically considered hypoxic, although many organisms experience negative effects at higher oxygen levels. (C) Relationship between mean daily minimum DO and mean daily minimum pH at the 29 sites with at least 20d of monitoring for both parameters.

Far less is known about the effects of diel-cycling hypoxia, which is common in nearshore coastal environments, and occurs at shallow depths that are refuges from deeper water oxygen depletion and are important habitat for a wide range of organisms. Diel-cycling hypoxia occurs in the photic zone during night and early morning hours when respiration exceeds oxygen production by photosynthesis, and is associated with high algal or macrophyte biomass [9]. Because the severity of night-time oxygen depletion depends on a number of factors (e.g., flushing, nutrient loading, daytime cloud cover) whose spatial patterns vary independently, considerable spatial variation in the severity and frequency of diel-cycling hypoxia can occur within a single system [9,10] (this paper: Fig. 1 and S1 Fig.). Field monitoring in tidal creeks and coastal bays of the mid-Atlantic coast of the United States has demonstrated that DO concentrations in a single 24-hour period can range from 0 (anoxia) to over 15 mg l^-1^, and while ephemeral, hypoxia may occur daily for extensive periods during the summer months [10,11].

Variation in the physical environment potentially influences disease processes through differential effects on pathogens and hosts [12,13]. Environmental conditions can affect pathogen proliferation and transmission through direct effects on the pathogen itself or on intermediate hosts [14,15]. In addition, conditions that compromise host responses to pathogens can result in increased numbers of infected individuals and proliferation of parasites within hosts [16,17]. Changes in environmental conditions, whether natural or human-caused, can therefore alter the landscape patterns of host-pathogen distributions and interactions, and influence both spatial and temporal patterns and the severity of epizootics [18,19]. A consequence can be correlations in spatial patterns of physical factors in the environmental and spatial patterns of production and abundance of economically and ecologically important species.

Nutrient enrichment resulting in eutrophication of aquatic systems can profoundly change host-parasite interactions in fish and wildlife communities [12,20]. One concern supported by available data is that eutrophication can accelerate transmission and magnify the pathology of parasitic and infectious diseases [12,21] that directly threaten efforts to conserve, protect, and restore freshwater and coastal ecosystems. Yet, the linkages between nutrient enrichment and the dynamics of parasitic and infectious diseases remain poorly understood, in part due to variation among ecosystems in the expression of various symptoms of eutrophication, which may result in variation in patterns of disease risk.

The protistan parasite, *Perkinsus marinus*, is the causative agent of Dermo, which has contributed to dramatic declines in populations of the eastern oyster, *Crassostrea virginica* in Atlantic and Gulf Coast estuaries of North America, including Chesapeake Bay [22,23]. Transmission of the parasite does not require an intermediate host and occurs when oysters are exposed to waterborne *P*. *marinus* during feeding [24]. Adult oysters are at greater risk of *P*. *marinus* infections than juveniles [25], presumably because older, larger oysters have repeatedly encountered the parasite over time, and filter more water than small oysters, therefore ingesting more *P*. *marinus* cells [26]. Higher infection rates, more severe infections, and greater disease mortality occurs at temperatures above 20°C and salinities above 12 [27,28]. The eastern oyster is an ecologically and culturally important species in estuaries and nearshore coastal habitats of the eastern United States that has supported large commercial fisheries. In addition, oysters are prolific filter-feeders that play a critical role in the transfer of primary production to benthic secondary production [29], create habitat for finfish and invertebrate species [30–32], and contribute to denitrification [33].

Low DO is thought to increase oyster susceptibility to disease agents by reducing hemocyte production of reactive oxygen intermediates in response to stimuli [34]. Production of reactive oxygen intermediates at 2.0 mg l^-1^ and 25°C (~28% saturation, 1.5 kPa) is only about half that at 100% oxygen saturation (5.2 kPa) [34]. Laboratory studies also indicate that mortality from *P*. *marinus* infections is increased by continuous exposure to moderate hypoxia (2.9 mg l^-1^; [5]). Although these studies suggest a link between hypoxia, disease, and oyster mortality, oysters are rare in subpycnocline waters that have persistent hypoxia. It is possible that the effects of diel-cycling hypoxia would be proportional to the duration of exposure, but it is also possible that the duration of hypoxic conditions is too brief to have negative effects, that even brief exposures to low oxygen result in persistent reductions of immune responses, or that compensatory mechanisms during high oxygen phases make up for impairment during the low oxygen phase.

We used field and laboratory experiments to test the hypothesis that diel-cycling hypoxia increases oyster susceptibility to infection by *P*. *marinus*, with susceptibility defined in terms of acquisition and progression of *P*. *marinus* infections as well as disease-related mortality. First, we analyzed patterns of diel-cycling hypoxia in Chesapeake Bay. Second, field experiments evaluated whether disease status, mortality and growth in experimental oysters deployed at 14 locations in Chesapeake Bay varied spatially with the frequency or severity of diel-cycling hypoxia. Finally, we tested these same end points, abundance of waterborne *Perkinsus*, and clearance of phytoplankton by oysters in laboratory experiments that could control for spatial variation in pH, temperature and prey.

## Materials and Methods

### Ethics Statement

Permission was acquired for use of field sites and for field experiments from Maryland Department of Natural Resources and property owners. The study did not involve endangered or protected species, human subjects, or vertebrate animals.

### Field Monitoring

We used dissolved oxygen and other water quality from the Maryland Department of Natural Resources (MD-DNR) Shallow Water Monitoring Program http://mddnr.chesapeakebay.net/eyesonthebay (last accessed 24 June 2014) and, for field experiments, also from the Virginia Estuarine and Coastal Observing System (web2.vims.edu/vecos; last accessed 24 June 2014) [11,35]. Yellow Springs Instruments model 6600 sondes were deployed in 1–2 m of water and recorded DO, temperature, salinity, *in vivo* fluorescence (as a measure of chlorophyll *a*), and in most cases pH (calibrated with NBS standards) and turbidity, at 15 minute intervals 24h d^-1^. Sondes were replaced every two weeks with freshly calibrated meters. For analyses of patterns of diel-cycling hypoxia in potential oyster habitat, we included all sites monitored by MD-DNR and partners in MD waters during 2001–2011 with minimum mean July-August salinity ≥7.0 and near-bottom sensors (0.3 or 0.5 m above the bottom). Further details on methods and metadata can be found at http://mddnr.chesapeakebay.net/eyesonthebay/stories.cfm (Last accessed 24 June 2014).

### General Infection Metrics and Assays

Ray’s fluid thioglycollate assay (RFTM) on rectal tissue [36] was used to estimate infection prevalence (the percentage of oysters testing positive for *P*. *marinus*) and intensity (a measure of proliferation of *P*. *marinus* cells within oyster tissue) for both field and laboratory experiments. RFTM can miss very light infections [37,38], but is the preferred method for screening large numbers of oysters because of the low cost and speed with which large samples can be processed; RFTM is the standard method used in monitoring programs in Chesapeake Bay (e.g.[39]). Oysters referred to as ‘uninfected’ tested negative using the RFTM assay; the effect of missing infections below detection limits on study conclusions are considered in the Discussion.

The change in disease prevalence between the start and conclusion of the experiment was used as an index of disease acquisition at each site. Mean infection intensity (mean Mackin score of individuals testing positive for *P*. *marinus*) was used as a primary measure of disease progression. Scores were assigned using the modified Mackin scale [40], which ranges from 0.5 (*P*. *marinus* cells rare in the RFTM preparation) to 5 (a heavy infection) based on the density and distribution of *P*. *marinus* cells within the tissue being examined. We also used the prevalence of moderate-to-heavy infection intensities (Mackin scores ≥2) as a measure of infection progression for experiments in which prevalence was increased by treatments but intensity was not; inclusion of individuals with newly acquired, low intensity infections can obscure the relationship between mean intensity and infection progression. For clarity we refer to prevalence calculations based on all oysters as ‘total prevalence’ and those based on individuals with medium-to-heavy infections as ‘MHprevalence’. We also used quantitative PCR (qPCR; [41]; see S2 Methods) to specifically quantify *P*. *marinus* infection intensity in a subsample of 1yo (year-old) oysters from 2009 experiments in order to relate RFTM scores to *P*. *marinus* density in oyster tissue. Additional experiment-specific *P*. *marinus* methods and measures are described below.

### Field Experiments

**Fig 2:**
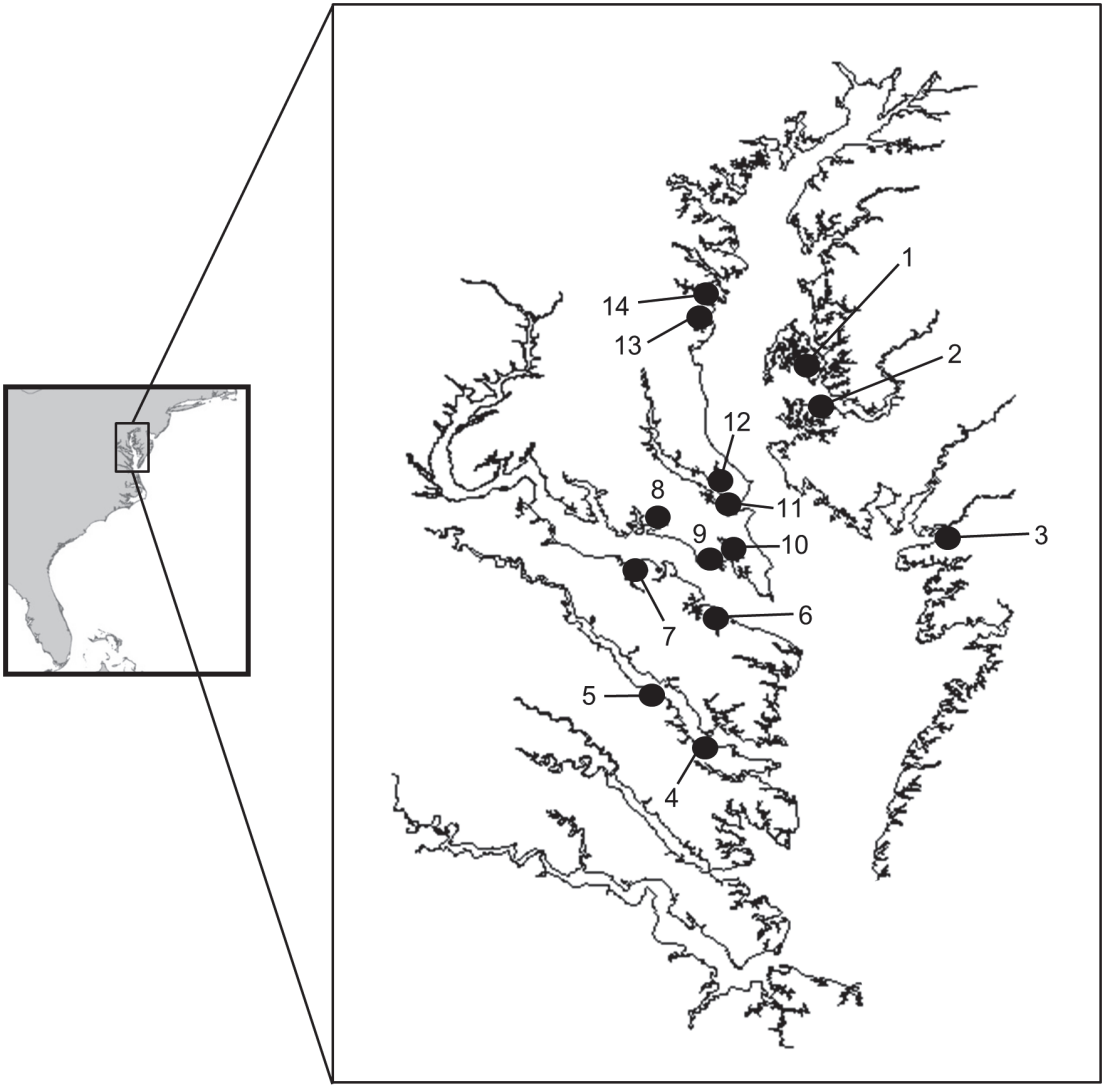
Oyster deployment locations for field experiments. (1) Choptank R. at Mulberry Pt., (2) Choptank R. at Univ. MD Horn Pt. Laboratory, (3) Little Monie Creek, (4) lower Rappahannock R., (5) middle Rappahannock R., (6) West Yeocomico R., (7) Nomini Creek, (8) Potomac R. at Breton Bay, (9) St. George’s Cr.(Potomac R.), (10) St. Mary’s R., (11) Patuxent R., (12) Cole’s Creek, (13) Rhode R., (14) Harness Creek (South R.). See S1 Table for GPS coordinates and year sampled.

**Experimental design.** Mixed stocks of ‘initially-infected’ and ‘initially uninfected’ oysters were deployed in the mesohaline portion of Chesapeake Bay at 5 sites in 2008 and 9 sites in 2009 to examine whether the prevalence and intensity of *P*. *marinus* infections increased with increasing severity and frequency of diel-cycling hypoxia (Fig. 2, S1 Table, S1 Methods). We also tested for effects on oyster mortality and growth. Sites were selected from the available pool of shallow water monitoring sites to include as broad a range of DO conditions as possible where *P*. *marinus* was already present and salinities were conducive to oyster growth and disease transmission. Interannual variability in rainfall limited our ability to predict environmental conditions *a priori*. Initially-uninfected oysters were used to assess acquisition and progression of *P*. *marinus* infections, oyster mortality and growth, and to predict the outcomes of restoration efforts in which putatively disease-free hatchery oysters are added to oyster reefs that contain diseased individuals. Initially-infected oysters had acquired *P*. *marinus* infections naturally in their source locations. They were deployed together with initially-uninfected oysters to provide a standardized source of *P*. *marinus* to uninfected individuals, and to test hypotheses about disease progression and mortality among naturally-occurring oyster populations in which *P*. *marinus* infections are already present.

One year-old oysters (1yo) purchased from an aquaculture facility on the mesohaline Choptank R. (Marinetics, Inc., Cambridge, MD, USA) were used as the initially-uninfected stock. RFTM assays indicated that these oysters were not infected with *P*. *marinus* (i.e., total prevalence = 0). During both years, we used a mixture of 2yo and 3yo oysters purchased from the same facility as our initially-infected stock. In 2008, initially-infected oysters also included adult-sized oysters collected from the middle Patuxent R., near the mouth of St. Leonard Cr., where naturally-occurring oysters were known to be heavily infected with *P*. *marinus*, to ensure the source of *P*. *marinus* 1yo oysters was adequate. Oysters from the Patuxent R. were not included in the initially-diseased group in 2009 because tests suggested that the hatchery oysters alone were an adequate source of *P*. *marinus*. Total prevalence and intensity of *P*. *marinus* infections in initially-infected oysters at the start of field experiments were 20% and 57%, and 1.0 and 1.5, respectively in 2008 and 2009 (see Table 1 for details on initial disease status and shell height). Shell height of 48 tagged oysters from each age class and site was measured at the beginning and end of field deployments.

**Table 1:** Correlations among water quality parameters at 36 shallow water monitoring sites in the Maryland portion of Chesapeake Bay.

Parameter	$\bar{X}$ dminDO	$\bar{X}$ dmin pH	$\bar{X}$ daily salinity	$\bar{X}$ dmax *IVF*
$\bar{X}$ dminDO		*R* = 0.70, *P*<0.0001	*R* = -0.22, *P* = 0.014	R* = -0.65, *P*<0.0001
$\bar{X}$ dmin pH	*R* = 0.66, *P* = 0.0001			
$\bar{X}$ daily salinity	*R* = -0.03, *P* = 0.89			
$\bar{X}$ dmax *IVF*	*R* = -0.69, *P*<0.0001			

Pearson correlations are reported except as noted with an * for Spearman Rank Correlation results. Values arranged horizontally use each year at a site as an independent value; results listed vertically combine multiple years at each site.

Oysters were suspended from docks or piers near to and at the exact same depth as the water quality sensors in polyethylene cages (61cm x 61cm x 20cm and with 2.3cm x 1.1cm mesh sides, bottom, and top) to exclude large predators. Cages were inspected every 2–3 weeks and replaced if biofouling threatened to restrict water exchange. Oysters were deployed between June 4 and June 13 in 2008, and between June 11 and 22 in 2009 and were retrieved both years during the first week of October.

In 2008, 480 oysters (160 1yo, 160 2+yo, 160 Patuxent R. oysters) were deployed at each location and subdivided into 8 cages at a ratio of 1:1:1 1yo hatchery oysters to 2–3yo hatchery oysters to Patuxent R. oysters. Oysters from the Patuxent were used only in 2008, served only as a disease source, and were not included in analyses of environmental effects. In 2009, the number of oysters deployed at each location was reduced to 300 (120 1yo, 180 2yo+ divided among 6 cages) because oyster mortality in 2008 was low. At the conclusion of the experiment, a random sub-sample of 24 oysters (3/cage in 2008, 4/cage in 2009) from each treatment (initially-uninfected, initially-infected) and site was tested for *P*. *marinus* infections. Subdividing oysters among several cages was done to limit weight of cages; the experimental unit for analyses was site, not cage.

**Data analysis.** Frequency of diel-cycling hypoxia was quantified as the percentage of days with minimum DO concentration ≤ 2.0 mg l^-1^. This threshold corresponds to the DO concentration at which Boyd and Burnett [34] observed suppression of oyster hemocyte function. Sites with frequent excursions to 2 mg l^-1^ also experienced daily minima of 3 mg l^-1^ more often, and were more likely to experience severe hypoxia. Severity of diel-cycling hypoxia was measured as the mean daily minimum DO concentration (in mg l^-1^) at each site.

We used general linear models (SAS Proc GLM; year random) to test for a relationship between the response variables (infection prevalence, infection intensity, mortality, growth), a continuous variable describing the frequency or severity of diel-cycling hypoxia, and year, which was included in the model to account for interannual variation in salinity, temperature, and the disease load of initially-infected oysters. In a second approach, we leveraged the high temporal resolution of the water quality data by regressing the response variables against a composite variable that measured the frequency or severity of diel-cycling hypoxia during days when minimum salinity was equal to or above potential threshold values (10, 11 or 12) at which Dermo epizootics typically occur. Candidate models for oyster growth also included chlorophyll *a* (as measured by *in vivo* fluorescence [IVF]). This approach does not consider selectivity of oyster feeding. Separate analyses were performed for initially-uninfected and initially-infected oysters. For all statistical tests, variables expressed in percent were transformed prior to analysis. All statistical analyses were performed in SAS (Version 9.2, SAS Institute Inc., Cary, NC, USA) and SigmaPlot (Version 12, Systat Software, Inc., San Jose, CA, USA). Best-fit models were selected on the basis of AICc values [42].

### Laboratory Experiments

During summer—early fall 2009 and 2010, we conducted laboratory experiments at SERC testing the effects of diel-cycling hypoxia on *P*. *marinus* infection acquisition and progression, growth, and mortality of 1- and 2–3yo eastern oysters. Oysters were purchased from Marinetics, Inc., and during 2009, belonged to the same cohorts as used for field deployments. Before use in experiments, and periodically during experiments, we measured oyster shell heights to the nearest mm and analyzed a subsample of oysters for *P*. *marinus* infections using standard RFTM assays [36]. As in field experiments, the goal was to use 1yo oysters with zero or near-zero total prevalence to test for effects of hypoxia on acquisition and progression of infections, and older oysters with a moderate total prevalence of light-to-moderate infections to serve as a source of infections and to test for effects of hypoxia on infection progression (additional information in S2 Methods).

**Fig 3:**
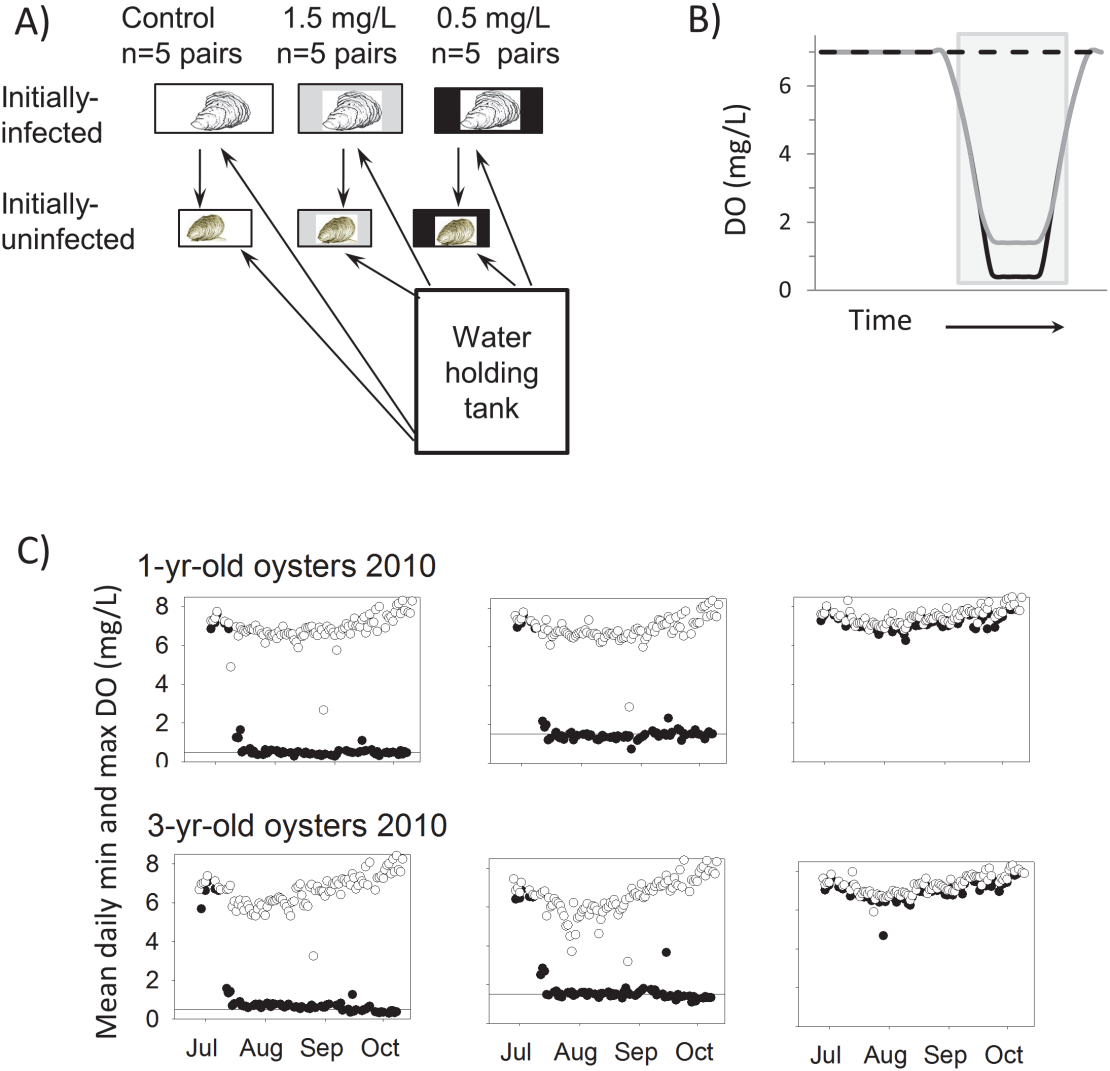
Laboratory experiment. (A) Experimental treatments. Arrows indicate direction of water flow. (B) Idealized dissolved oxygen cycle in experiments. Dashed black line = high DO control treatment; grey line = 1.5 mg l^-1^ treatment; black solid line = 0.5 mg l^-1^ treatment. The grey shaded box indicates the dark period. (C) Daily minimum (black circles) and maximum (open circles) dissolved oxygen concentrations measured in experiment tanks for each treatment in 2010. Horizontal lines indicate target DO concentrations in the 2 diel-cycling hypoxia treatments.

**2009 experiment.** Fifty-five individuals of each age-class of oysters were allocated to each of 5 replicate aquaria for each of 3 DO treatments (Fig. 3). Tanks were arranged in the room in a randomized block design in case room position affected results. Control (high DO) 37L aquaria containing 1yo oysters and 75L aquaria containing 2yo oysters were bubbled continuously with air. Mixes of N_2_ and air controlled with gas proportioners were used to cycle low-oxygen treatment tanks to minima of either 1.5 mg l^-1^ or 0.5 mg l^-1^. Thirty percent of water flow to 37L aquaria was pumped from 75L aquaria to provide a source of infection; all other water was supplied from the Rhode R. via the SERC seawater system at a volume equivalent to 9X tank volumes d^-1^. DO in aquaria with diel-cycling hypoxia ramped down from near saturation levels to target low DO over a 3 hour period, maintained minimum DO concentrations for 4 hours, and ramped back to near-saturation levels over a 3 hour period. Target DO minima were approached or achieved 4–5 days per week; tanks were bubbled only with air on weekends. In shallow Chesapeake Bay waters, severely hypoxic conditions do not typically occur 7 d wk^-1^ (see Results).

DO was measured with a YSI Professional Plus DO meter (Yellow Springs Instruments, Yellow Springs, OH, USA); pH was measured with an Oakton Acorn handheld meter. Subsamples of oysters from each tank were tested at approximately monthly intervals to estimate the intensity and prevalence of *P*. *marinus* infections. All remaining live oysters not used for disease assays were measured at the end of the experiment. In addition, we used quantitative PCR (qPCR; [41]; see Appendix B) to quantify waterborne *P*. *marinus* cells in individual aquaria and in exchange water on 8 dates as a measure of the effect of diel-cycling hypoxia on the release of *P*. *marinus* and as a measure of transmission risk for initially uninfected 1yo oysters [43,44].

**2010 experiment.** We repeated the same experimental treatments in 2010 but with several modifications. We supplemented phytoplankton supplied to experimental tanks by adding approximately 378 L/wk of *Chaetoceros muelleri* cultures to our water holding tanks, measured tissue wet weight as well as shell height, used 90 oysters per tank to allow for larger sample sizes for disease assays, and only sampled oysters during one midpoint sample period. We also used 3yo oysters for the older, infected oysters in experiments. Finally, we measured IVF with a Turner Designs 10AU fluorometer in experimental aquaria in at least 4 replicate tanks of each treatment at simulated dawn (after approximately 3 hours exposure to target oxygen concentrations) on 14 dates for each size class, as well as in 20L containers with oxygen concentrations matching those in experimental tanks as conditions returned to near-100% oxygen saturation following low oxygen exposure (Fig. 3b). A more complete examination of the relationship between diel-cycling DO and oyster clearance rates is presented in Clark [45]; here we present only data relevant to interpreting results of *P*. *marinus* acquisition and progression experiments.

**Data analysis for laboratory experiments.** Parameters measured on individual oysters within replicate tanks (e.g. oyster size, infection intensity) were analyzed as nested ANOVA using Proc GLM (SAS Version 9.2). Parameters yielding a single measurement per replicate tank (e.g., infection prevalence, DO) were analyzed as one-way ANOVA using Proc GLM with least square means comparisons testing *a priori* hypotheses that infection measures would be higher and filtration rates lower in the low DO treatments than in controls, and that effects in the 1.5 mg l^-1^ treatment would be less than in the 0.5 mg l^-1^ treatment. Prevalence data were logit transformed prior to analyses.

During 2010, ending total prevalence was high in all treatments, leaving little scope for detecting differences among treatments. Because 37L tanks received some water from larger aquaria containing infected oysters, in 2010 we also tested effects of DO treatment on prevalence and intensity of infections in 1yo oysters with an ANCOVA using mean infection intensity in the paired large aquaria as the covariate. We used a repeated measures MANOVA to test for treatment effects on chl *a* (calculated from IVF) measured in experimental aquaria.

## Results

### Field Monitoring

Thirty-six sites in Maryland Chesapeake Bay subestuaries and coastal bays were monitored for at least 1 summer during 2001–2012 with meters 0.3–0.5 m above the substrate and mean July-August salinity ≥7.0. Diel-cycling hypoxia occurred throughout the bay at depths equivalent to historical oyster habitat. During at least 50% of days in July-August, DO dropped to or below 2.0 mg l^-1^ at 10 sites, below 1.5 mg l^-1^ at 7 sites, and below 0.5 mg l^-1^ at 3 sites (Fig. 1a). Mean daily minimum ($\bar{X}$ dmin) DO concentrations ranged from 0.82 ±0.12 (n = 58) mg l^-1^ in the St. Mary’s R. in 2008 to 6.34±0.22 (n = 24) in the Honga R. in 2009 (not shown), and varied among years (Fig. 1b). Highest $\bar{X}$ dmin DO among sites used in the field experiment was 5.28±0.08 (n = 61) at Mulberry Point in the Choptank River in 2008 (Fig. 1b). In analyses using all sites and years (n = 124), $\bar{X}$ dmin DO was positively correlated with $\bar{X}$ dmin pH (Fig. 1c), negatively correlated with mean salinity, and negatively correlated with mean daily maximum *in vivo* fluorescence (IVF: a measure of chlorophyll, Table 1). Relationships among physical variables were similar if all years at each site were averaged except that $\bar{X}$ dmin DO and mean salinity were not significantly correlated (Table 1).

### Field Experiments

**Physical Parameters and Chlorophyll a.** DO concentrations in the 14 shallow subtidal study sites used in field experiments (Fig. 2) exhibited a diel cycle throughout the experimental period (June-September), with daily minima generally occurring between 0600 and 0800 hrs and daily maxima between 1400 and 1800 hrs. Diel patterns in DO concentrations varied among sites; daily DO minima periodically fell below 1 mg l^-1^ at some sites but remained above 4 mg l^-1^ at the sites with highest daily minimum DO concentrations (Fig. 1b and S1 Fig.). Temperatures conducive to *P*. *marinus* proliferation (≥ 20°C) prevailed throughout the majority of the experimental period at all study sites and during both years (S2 Fig.). Salinity generally increased throughout the experimental period, but the onset and duration of salinities most favorable for the proliferation of *P*. *marinus* (salinity ≥ 10 to 12) varied among study sites and years (S2 Fig.). In 2008, salinity at most sites did not reach 10 until August, whereas in 2009, some sites were above this threshold for nearly the entire study period.

Both average daily mean ($\bar{X}$ dmean) and $\bar{X}$ dmin DO at these sites were positively correlated with $\bar{X}$ dmin pH (R = 0.55, *P* = 0.049 and R = 0.58, *P* = 0.039, respectively), but were not significantly correlated with $\bar{X}$ dmean IVF, $\bar{X}$ dmean temperature or $\bar{X}$ dmean salinity (all *P*>0.45). $\bar{X}$ dmin pH ranged from 6.8 at Little Monie Cr. to 7.9 at the lower Rappahannock R. site. Below we analyze for effects using DO as the independent variable, but consider the potential role of pH in the Discussion. $\bar{X}$ dmin pH was negatively correlated with IVF (R = -0.65, p = 0.017) and temperature (R = -0.74, p = 0.004), and positively correlated with salinity (R = 0.69, p = 0.008). $\bar{X}$ dmean and $\bar{X}$ dmin DO were strongly correlated (R = 0.94, *P*<0.0001).

**Fig 4:**
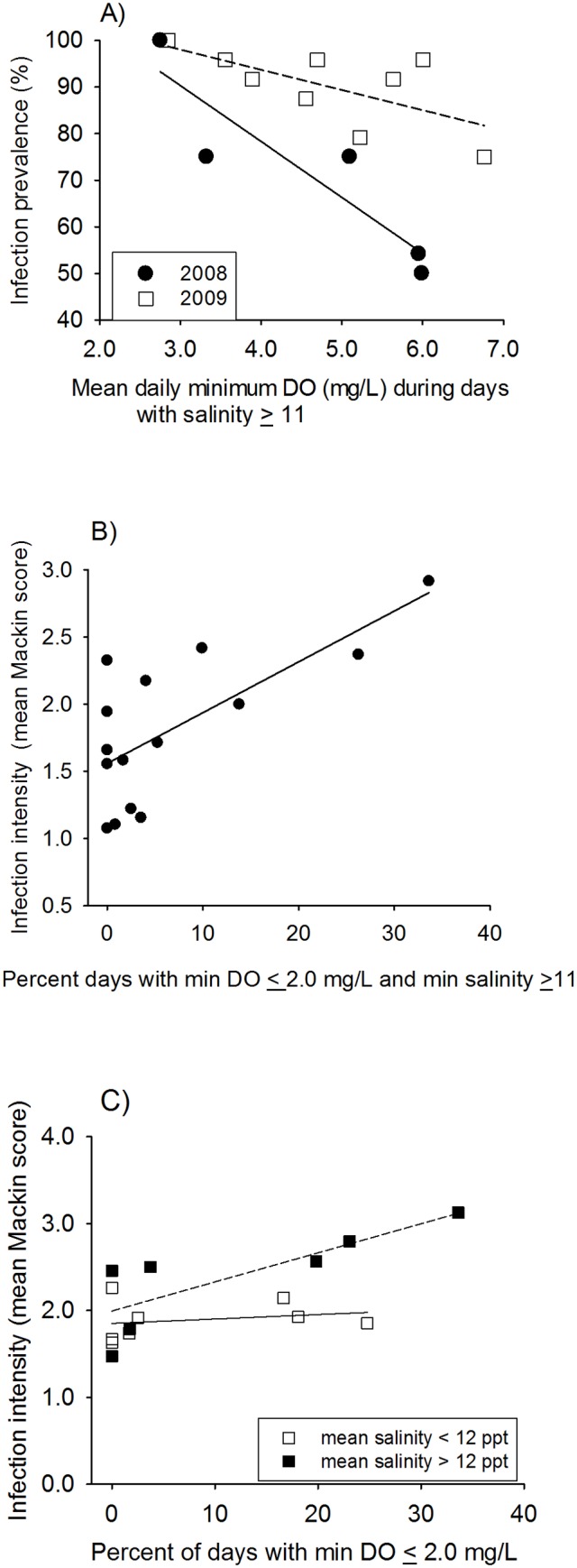
Field experiment. Relationships between hypoxia and (A) prevalence and (B) intensity of *P*. *marinus* infections in initially-uninfected oysters, and (C) intensity of infections in initially-infected oysters.

**Oyster Disease.** Final total *P*. *marinus* prevalence in initially-uninfected (1yo) hatchery oysters ranged from 50% (Choptank R.-Horn Pt. Laboratory, 2008) to 100% (Harness Cr., 2008; St. Mary’s R., 2009), decreased significantly with increasing $\bar{X}$ dmin DO, and differed between years (Table 2, Fig. 4a). Variation among sites in final total prevalence was best explained by a general linear model including $\bar{X}$ dmin DO during days with minimum salinity ≥ 11 (hypoxia-salinity effect; *F*_*1*,*9*_ = 15.36, *P*<0.004), year (*F*_*1*,*9*_ = 1.66, *P* = 0.23), salinity (*F*_*1*,*9*_ = 2.02, *P* = 0.19), and the hypoxia-salinity×year interaction (*F*_*1*,*9*_ = 3.91, *P* = 0.08), although only the hypoxia-salinity main effect was statistically significant. Final total *P*. *marinus* prevalence among initially-infected hatchery oysters ranged from 71% (Choptank R.-Horn Pt. Laboratory, 2008) to 100% (Harness Cr., 2008; St. Mary’s R., Patuxent R., and lower Rappahannock R., 2009), but was not significantly related to either diel-cycling hypoxia or salinity (Table 2, *P* > 0.11 for all candidate models). Only 3 deployments had prevalence levels <75%, and all of these sites had $\bar{X}$ dmin DO concentrations >5.2 mg l^-1^ on days when salinity equaled or exceeded 11. Final prevalence at all other sites was >88%, leaving little scope for detecting environmental effects.

**Table 2:** Results of ANOVAs testing effects of mean daily minimum DO and year on the prevalence and intensity of *P*. *marinus* infections at the end of the field experiment.

Initial disease treatment	Disease metric	Test	***df***	***F***	***P***
uninfected	prevalence	model	2,11	6.34	0.015
		$\bar{X}$ dmin DO	1,11	7.36	**0.020**
		year	1,11	8.13	0.016
uninfected	intensity	model	2,11	3.14	0.084
		$\bar{X}$ dmin DO	1,11	4.69	**0.053**
		year	1,11	2.91	0.116
infected	prevalence	model	2,11	1.11	0.363
		$\bar{X}$ dmin DO	1,11	1.51	**0.244**
		year	1,11	1.20	0.297
infected	intensity	model	2,11	5.05	0.022
		$\bar{X}$ dmin DO	1,11	8.75	**0.013**
		year	1,11	4.48	0.058

Nonsignificant interaction terms were removed from models; only final models are presented below. *P* values for DO effects are bolded for emphasis.

Final infection intensity among initially-uninfected oysters ranged from 1.1 (Choptank R.-Mulberry Pt., 2008) to 2.9 (St. Mary’s R., 2009). Infection intensity tended to be affected by $\bar{X}$ dmin DO but not year (Table 2), and was best explained by the frequency of diel-cycling hypoxia during periods with salinity 12 or greater (*F*_*1*,*12*_ = 14.2, P = 0.003; Fig. 4b). Final *P*. *marinus* infection intensity among initially-infected oysters ranged from 1.5 (Yeocomico R., 2008) to 3.1 (St. Mary’s R., 2009), decreased significantly with increasing $\bar{X}$ dmin DO, and tended to differ between years (Table 2). The model that best explained variation in final infection intensity of initially uninfected oysters included the frequency of diel-cycling hypoxia and a categorical variable that separated experimental sites into two groups based on whether mean salinity during the study period was < 12 or ≥ 12 (nearly identical results were found using a salinity of 11 as the cutoff). Infection intensity increased with the frequency of diel-cycling hypoxia, but only at sites in the higher salinity category (hypoxia F = 7.27, P = 0.021, hypoxia×salinity F = 9.05, P = 0.012, overall model *F*_*2*,*11*_ = 11.14, *P* = 0.002; Fig. 4c).

**Oyster Mortality and Growth.** Cumulative mortality ranged from 5% (upper Rappahannock R., Potomac R.-Breton Bay, Potomac R.-St. George’s Cr.; 2009) to 26% (Harness Cr., 2008) in initially-uninfected oysters, and from 4% (Choptank R.-Horn Pt. Laboratory, 2008) to 58% (lower Rappahannock R., 2009) in initially-infected oysters. Mortality of initially-infected oysters was significantly lower in 2008 (9.6%) than in 2009 (22.4%) (*t*-test, unequal variance, *df* = 14, *t* = −2.48, *P* = 0.03), but was not associated with salinity or the frequency or severity of diel-cycling hypoxia for either initially-uninfected or initially-infected oysters even when year effect was included in models (P > 0.10 for all comparisons).

**Fig 5:**
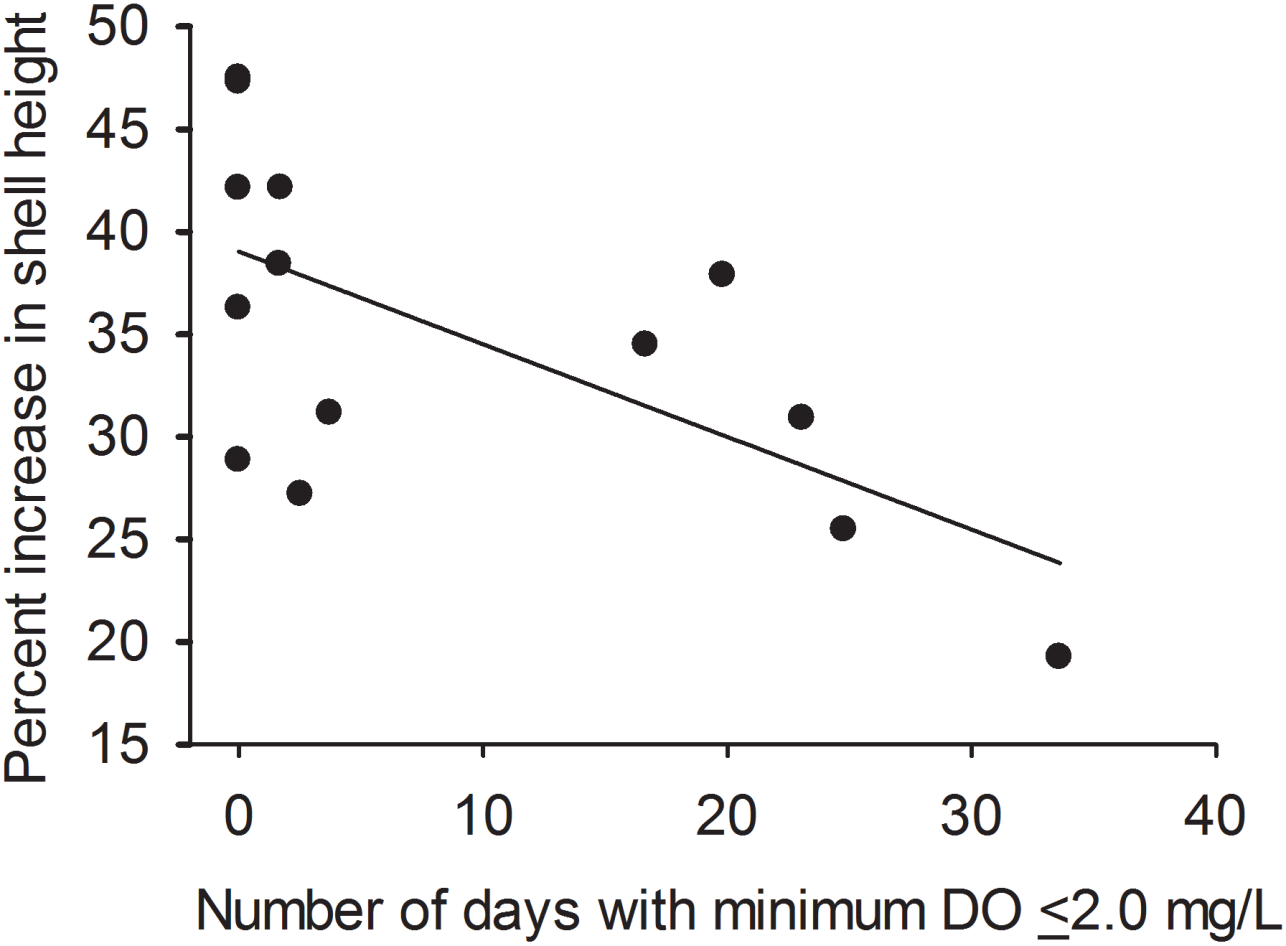
Percent increase in the shell height (*GROW*) of initially-uninfected (1yo) oysters versus the percentage of days with minimum DO concentration ≤ 2.0 mg l^-1^ (*HYP*) across 14 experiment deployment locations in Chesapeake Bay during 2008 and 2009. Regression line: *GROW* = 39.03–0.45*HYP, *r* ^2^ = 0.41, *P* = 0.01, *n* = 14.

Percent increase in shell height for initially-uninfected oysters ranged from 19% (St. Mary’s R., 2009) to 47% (Choptank R.-Mulberry Pt., 2008) and declined significantly across sites as the frequency of diel-cycling hypoxia increased (*R* ^*2*^ = 0.41, *P* = 0.01, n = 14; Fig. 5). The effect of food concentration (as indicated by chl-*a* concentration) on shell growth was not significant (*P* = 0.24). Percent increase in shell height for initially-infected oysters ranged from 2% (Little Monie Cr., 2008) to 18% (Patuxent R., 2009) and was not associated with frequency or severity of diel-cycling hypoxia, salinity, diel-cycling during salinities above potential thresholds, or chl *a* concentration (*P* ≥ 0.20 for all models).

### Laboratory Experiments

Exposure to diel-cycling hypoxia increased total *P*. *marinus* infection prevalence in *C*. *virginica* in both 2009 and 2010 experiments, and MHprevalence (prevalence of moderate-to-heavy infections) in 2009. Variation in results among years may have reflected variation in physical conditions, initial infection rates of 1yo oysters, and the size, infection prevalence and infection intensity of older oysters used in experiments. Details of physical measurements and sizes of oyster are provided in Table 3, and tests of initial sizes and disease status are in S1 Results. Laboratory experiment treatments did not substantially affect *P*. *marinus* infections, decrease growth, or increase mortality in 2–3 year old oysters (Table 4, S3 Fig.); results for these oysters are therefore also presented in S1 Results.

**Table 3:** Physical conditions, sizes of oysters, and mortality in 2009 and 2010 laboratory experiments [X ± SE (n)].

	40 L aquaria, 1 year-old oysters			80L aquaria, 2 and 3 year-old oysters		
	Control	1.5 mg l^-1^	0.5 mg l^-1^	Control	1.5 mg l^-1^	0.5 mg l^-1^
**2009**						
Daily maximum DO (mg l^-1^)	7.30±0.03 (265)	6.52±0.07 (265)	6.61±0.08 (266)	7.18±0.03 (265)	6.46±0.07 (265)	6.28±0.08 (265)
Daily minimum DO (mg l^-1^)	6.68±0.07 (265)	1.46±0.02 (265)	0.66±0.08 (265)	6.48±0.08 (265)	1.63±0.02 (265)	0.78±0.01 (265)
Temperature (°C)	25.0±0.1 (285)	25.0±0.1 (285)	25.0±0.1 (285)	25.2±0.2 (285)	25.3±0.2 (285)	25.3±0.2 (285)
pH	7.52±0.02 (65)	7.44±0.01 (65)	7.52±0.02 (65)	7.48±0.02 (65)	7.40±0.02 (65)	7.44±0.02 (65)
Salinity	11.3±0.1 (186)	11.2±0.1 (196)	11.3±0.1 (187)	11.3±0.1 (188)	11.2±0.09 (196)	11.2±0.04 (197)
Starting shell height (mm)	50.5±0.4 (275)	51.4±0.4 (275)	50.2±0.4 (275)	76.6±0.6 (275)	76.8±0.7 (275)	77.5±0.8 (275)
Ending shell height (mm)	54.7±0.8 (102)	53.7±0.7 (108)	53.9±0.7 (100)	73.1±0.9 (97)	73.0±1.0 (108)	73.7±1.2 (94)
Total mortality (%)	10.9±3.7 (5)	10.9±4.5 (5)	9.1±3.8 (5)	12.4±1.0 (5)	8.7±1.8 (5)	9.5±1.1 (5)
**2010**						
Daily maximum DO (mg l^-1^)	7.53±0.02 (321)	6.91±0.03 (321)	6.89±0.04 (321)	7.35±0.03 (320)	6.54±0.06 (320)	6.65±0.05 (320)
Daily minimum DO (mg l^-1^)	7.16±0.02 (321)	1.47±0.03 (321)	0.50±0.01 (321)	7.00±0.03 (321)	1.53±0.02 (320)	0.64±0.01 (320)
Temperature (°C)	26.0±0.11 (268)	26.0±0.11 (273)	26.1±0.11 (269)	26.5±0.12 (271)	26.4±0.13 (279)	26.5±0.12 (267)
pH	7.79±0.03 (20)	7.82±0.03 (20)	7.96±0.03 (20)	7.71±0.03 (20)	7.76±0.03 (20)	7.94±0.03 (20)
Salinity	11.8±0.1 (247)	11.8±0.1 (247)	11.8±0.1 (245)	11.8±0.1 (248)	11.8±0.1 (259)	11.8±0.1 (248)
Starting shell height (mm)	46.4±0.4 (450)	45.8±0.4 (450)	45.5±0.4 (450)	78.9±0.3 (450)	78.5±0.3 (450)	78.6±0.3 (450)
Ending shell height (mm)	46.2 ±0.4 (267)	45.2 ±0.4 (277)	44.4 ±0.4 (272)	79.2±0.5 (194)	77.9±0.5 (203)	79.1±0.5 (216)
Ending tissue wet weight (g)	1.07±0.04 (150)	0.92±0.04 (150)	0.93±0.05 (150)	5.84±0.14 (150)	5.15±0.14 (150)	6.09±0.05 (150)
Total mortality (%)	6.2±1.3 (5)	7.2±3.3 (5)	7.1±1.8 (5)	24.2±1.6 (5)	21.1±0.9 (5)	18.4±1.7 (5)

Means were calculated from daily mean, minimum or maximum values of replicate measurements within each aquarium averaged over all dates on which DO concentrations were manipulated. Because of low variation among aquaria of similar sizes, temperature and salinity were not always measured in all aquaria each day. In 2010, pH was measured in all tanks near the end of the low plateau period on 4 dates.

**Table 4:** Results of laboratory experiment statistical analyses.

Year	Initial disease status	Disease metric	Test	***df***	***F***	***P***
2009	uninfected	Total prevalence	diel-cycling hypoxia	2,12	9.19	0.004
		MHprevalence	diel-cycling hypoxia	2,12	3.72	0.055
		intensity	diel-cycling hypoxia	2,11*	0.15	0.277
		mortality	diel-cycling hypoxia	2,12	0.08	0.927
		shell growth	diel-cycling hypoxia	2,12	0.66	0.534
2009	infected	Total prevalence	diel-cycling hypoxia	2,12	0.05	0.954
		MHprevalence	diel-cycling hypoxia	2,12	0.03	0.972
		intensity	diel-cycling hypoxia	2,12	0.15	0.861
		mortality	diel-cycling hypoxia	2,12	0.98	0.403
		shell growth	diel-cycling hypoxia	2,12	0.53	0.600
2010	uninfected (ANOVA)	Total prevalence	diel-cycling hypoxia	2,12	0.03	0.720
		MHprevalence	diel-cycling hypoxia	2,12	0.05	0.95
		intensity	diel-cycling hypoxia	2,12	0.56	0.587
		mortality	diel-cycling hypoxia	2,12	0.08	0.924
		shell growth	diel-cycling hypoxia	2,12	13.81	0.001
		wet weight growth	diel-cycling hypoxia	2,12	4.13	0.043
	uninfected (ANCOVA)	Total prevalence	Model	7,7	5.20	0.02
			Diel-cycling hypoxia	2,7	5.97	0.031
			*P*. *marinus* density in paired tank	1,7	15.24	0.006
			Room position (block)	4,7	3.26	0.083
	infected	Total prevalence	diel-cycling hypoxia	2,12	4.17	0.042
		MHprevalence	diel-cycling hypoxia	2,12	0.13	0.881
		intensity	diel-cycling hypoxia	2,12	0.85	0.454
		mortality	diel-cycling hypoxia	2,12	3.98	0.047
		shell growth	diel-cycling hypoxia	2,12	1.42	0.280
		wet weight growth	diel-cycling hypoxia	2,12	0.79	0.477

One-way ANOVA was used to test effects of diel-cycling hypoxia on total prevalence and MHprevalence of *P*. *marinus* infections, as well as oyster mortality in both years. Nested ANOVA was used to test for effects on infection intensity and oyster growth. In addition ANCOVA was used to test for effects of diel-cycling hypoxia on total prevalence and growth in 1yo oysters for the 2010 experiment. For 3yo oysters in 2010, significant DO effects do not provide evidence for a negative effect of diel-cycling hypoxia; growth in the 0.5 mg l^-1^ treatment did not differ from that in controls, and mortality was higher in controls than in the 0.5 mg l^-1^ treatment.

*Control replicate with zero prevalence was not included in intensity analysis, resulting in a lower df.

**Fig 6:**
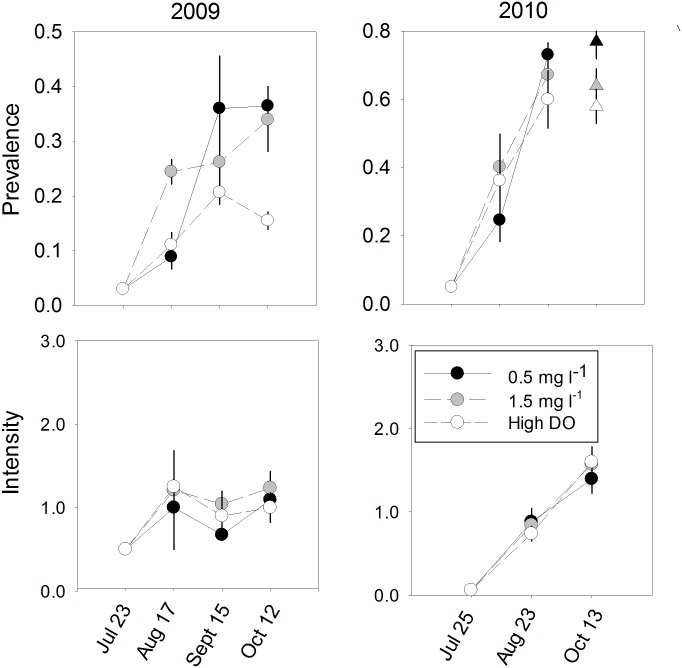
Prevalence and intensity of *P*. *marinus* infections in 1yo oysters in laboratory experiments (mean ± SE). Circles are means calculated from unadjusted data; triangles for 2010 1yo oysters show 13 October lsmeans for data adjusted for infection intensity in paired tanks containing 3yo oysters. Only infected individuals (i.e., Mackin score of 0.5 or greater) were included in intensity calculations.

**2009 Experiments.** Diel-cycling hypoxia led to increased total prevalence and a trend toward increased MHprevalence of *P*. *marinus* infections, but mean intensity of infections in 1yo oysters did not vary among treatments (Table 4, Fig. 6). By the end of the experiment, *P*. *marinus* total prevalence was nearly three times as high in diel-cycling treatments as in controls, averaging 34 and 36% in treatments with nominal daily minima of 1.5 and 0.5 mg l^-1^, respectively, compared to 12% in high oxygen controls. Planned contrasts (LSmeans) indicated that the difference between both diel-cycling hypoxia treatments and controls was statistically significant (both P<0.01). MHprevalence, which would measure proliferation of the parasite within oysters, averaged 10.8±3.4% and 9.5±2.7% in the 1.5 and 0.5 mg l^-1^ treatments, respectively, compared to 1.3±1.3% in high oxygen controls (0.5 mg l^-1^ vs control *P* = 0.032, 1.5 mg l^-1^ vs control *P* = 0.040). Mean intensity of infections in 1yo oysters that were infected at the end of the experiment ranged only from 1.0 to 1.2 across the three DO treatments.

There were no differences in mortality among DO treatments (Tables 3 and 4). Over the course of the experiment, mortality ranged from 9.1% in the 0.5 mg l^-1^ treatment to 10.9% in the 1.5 mg l^-1^ and high oxygen controls, respectively, for 1yo oysters. There was also no effect of DO treatment on ending oyster shell height (Tables 3 and 4).

**2010 Experiments.** As in 2009, final total prevalence varied among treatments, but total prevalence in all treatments was higher and DO treatment effects were less pronounced (Fig. 6, Table 4). Simple one-way ANOVA indicated that the variation in total prevalence, ranging from 59.8±8.4% in controls to 73±3.5% in the 0.5 mg l^-1^ cycling tanks, did not vary significantly among treatments. However, because of the paired design of the experiment, the intensity of *P*. *marinus* infections in 3yo oysters could be used as a measure of exposure risk to infective *P*. *marinus* cells by 1 yo oysters, and as a covariate in analyses. *P*. *marinus* intensity and MHprevalence in 3yo donor oyster tanks did not differ substantially among DO treatments (Table 4, S1 Results), The measure of infection risk used in this analysis was based on the number of *P*. *marinus* cells/g of gill and mantle tissue calculated from the relationship between Mackin scale intensity and *P*. *marinus* cell density using qPCR analyses of oyster tissue (S2 Methods). The analysis using this ANCOVA approach and block (room position) effect, indicated a significant effect of DO treatment, the estimated *P*. *marinus* cell density in oysters in the paired donor tank, and a trend towards a block effect. Planned LSmeans comparisons indicated that total prevalence was significantly higher in the 0.5 mg l^-1^ treatment than in controls (*P* = 0.011), and showed a trend toward being higher in the 1.5 mg l^-1^ treatment than in controls (*P* = 0.061). Final infection intensity in infected 1yo oysters and MHprevalence did not vary significantly among treatments; use of more complex models, as above, yielded substantially the same result. Intensity ranged from 1.40±0.2 in the 0.5 mg l^-1^ treatment to 1.60±0.2 in high DO controls; MHprevalence ranged from 27.3±6.0% in the 0.5 mg l^-1^ treatment to 30.3±8.8% in high DO controls.

DO treatment negatively affected growth of 1yo oysters (Tables 3 and 4). Differences among treatments were extremely small, but LSmeans comparisons indicated that final shell heights of oysters in the 0.5 mg l^-1^ were significantly smaller than shell heights of controls (*P* = 0.004). We analyzed final weight using both a simple nested ANOVA on all oysters remaining at the end of the experiment, and with an ANCOVA using infection intensity for oysters assayed with RFTM. Wet weight of 1yo oysters varied significantly among DO treatments (Table 4). LSmeans comparisons indicated that wet weight of oysters in both the 0.5 mg l^-1^ and 1.5 mg l^-1^ treatments averaged lower than those in controls (*P* = 0.0410 and *P* = 0.024, respectively). Results were similar when infection intensity was included as a covariate. DO treatment did not affect mortality of 1yo oysters (Tables 3 and 4).

**Fig 7:**
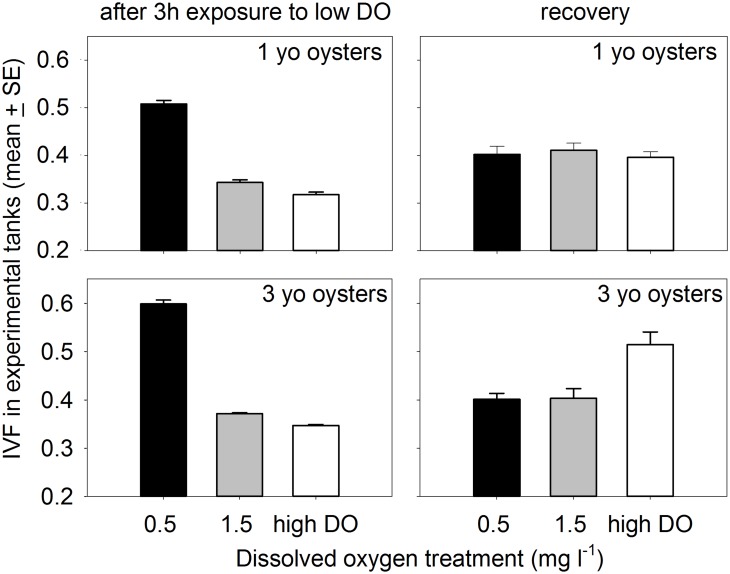
IVF measurements in 2010 experimental aquaria during the low DO phase of the cycle, and in separate 20-L containers towards the end of the recovery phase. For measurements during the low DO cycle, multiple measurements in tanks are averaged and the graphed data represent the grand means of individual tank means.

**Oyster Filtration.** Repeated measures analyses (SAS Proc Mixed) of measurements taken after approximately 3 hours at target oxygen concentrations indicated that IVF varied among DO treatments (*F*_*2*,*12*_ = 269.6, *P*<0.0001 for 1yo oysters, *F*_*2*,*12*_ = 863.4, *P*<0.0001 for older age class). Lsmeans comparisons indicated that all three DO treatments were distinct (all *P*<0.02) for both 1yo and older oysters. For both age classes, IVF in the 0.5 mg l^-1^ treatment averaged more than twice that in high oxygen controls (Fig. 7) indicating that clearance rates at the lowest oxygen concentrations tested were substantially reduced during exposure to the low oxygen phase of the daily cycle. Differences between the 1.5 mg l^-1^ and high oxygen controls were smaller, but statistically significant. The magnitude of difference between clearance rates in the 0.5 mg l^-1^ treatment and controls did not change during the course of the experiment with repeated exposure to hypoxia [45].

The filtration rate experiment that was conducted as aquaria returned towards normoxia, indicated that recovery from exposure to low oxygen was rapid even after 4 weeks of exposure to fluctuating oxygen concentrations. IVF in containers with 1yo oysters was similar for all DO treatments (one-way ANOVA *F*_*2*,*12*_ = 0.46, *P* = 0.64). In addition, IVF in containers with 3yo oysters previously held at 0.5 mg l^-1^ or 1.5 mg l^-1^ DO was significantly lower (i.e., clearance was higher) than in containers with control oysters (one-way ANOVA model *F*_*2*,*12*_ = 9.85, *P* = 0.003; LSmeans comparisons both low DO treatments vs controls: *P*≤0.005; Fig. 7) suggesting compensatory respiration and increased resultant filtration rates following exposure to severe hypoxia.

**Fig 8:**
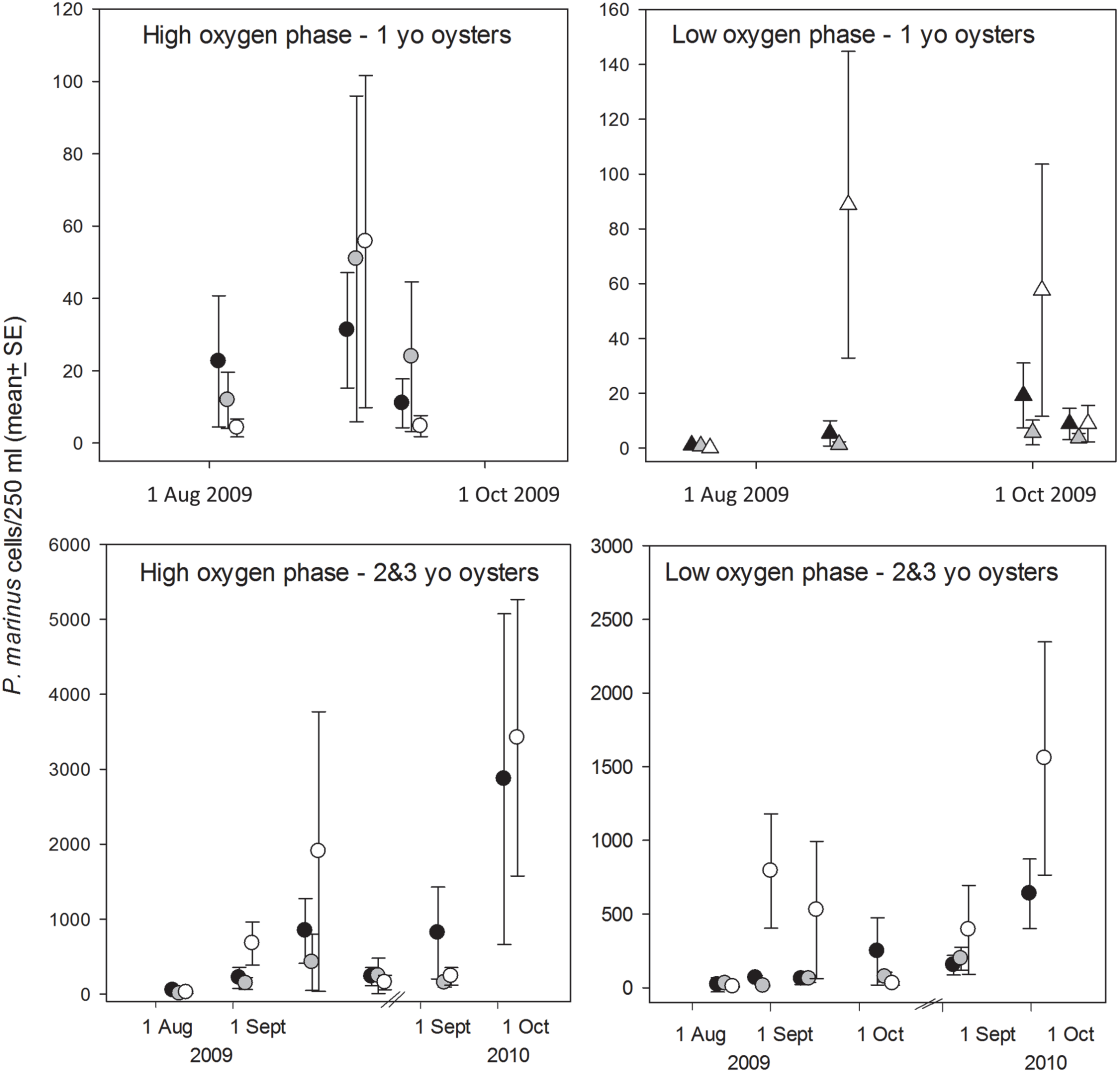
Densities of waterborne *P*. *marinus* cells in experimental aquaria based on qPCR assays. Symbols: black = 0.5 mg l^-1^ tanks; grey = 1.5 mg l^-1^ tanks; white = high oxygen control tanks.

**Waterborne *P*. *marinus* in Aquaria.** Waterborne *P*. *marinus* cell densities estimated with qPCR assays yielded highly variable results and did not provide support for an effect of diel-cycling DO on release of *P*. *marinus* from infected oysters (Fig. 8). In fact, during the low-oxygen phase of the diel-cycle, the parasite tended to be most abundant in control tanks on dates with the largest differences among treatments. We were unable to test all tanks each time we sampled, and therefore examined differences at individual time points with the acknowledgement that these tests are not truly independent and that the large number of tests increases the probability of a type 1 statistical error. Out of a total of 19 comparisons, only 3 indicated statistically significant differences among treatments (1-way ANOVA, P<0.05), and two of these were on the dates (25 and 26 August 2009) with lowest measured waterborne *P*. *marinus* cell densities. In tanks with initially infected oysters, *P*. *marinus* densities were significantly lower in tanks cycling to 1.5 mg l^-1^ DO than in controls tanks during the high-oxygen phase of the diel cycle on 26 August 2009 (F_2,6_ = 9.04, P = 0.032). In tanks with initially uninfected oysters, *P*. *marinus* densities were significantly lower in control tanks than in either hypoxic treatment during the low-oxygen phase on 25 August 2009 (F_2,6_ = 9.04, P = 0.027), and *P*. *marinus* densities were significantly lower during the low oxygen phase in both low-oxygen treatments than in control tanks on 9 September 2009 (F_2,12_ = 9.04, P = 0.004). A comparison of water supply tanks and aquaria containing initially-infected oysters sampled on the same date and phase of the cycle, indicated that *P*. *marinus* densities in holding tanks were only 3.4 ±1.7% (n = 6) that in experimental aquaria. These results indicate that the initially-infected experimental oysters were the primary source of *P*. *marinus* supplied to initially-uninfected oysters.

## Discussion

Results of this study indicate that exposure to diel-cycling hypoxia can increase the acquisition and progression of *P*. *marinus* infections in eastern oysters. In the field, both the acquisition of new infections in 1yo oysters, and the progression of infections in 1yo and older oysters, increased with increasing exposure to diel-cycling hypoxia. Laboratory experiments that helped isolate the effect of DO from those of pH, food and salinity, also indicated that brief, but frequent, periods of exposure to low DO concentrations can increase the acquisition of *P*. *marinus* infections by *C*. *virginica* oysters, and proliferation of the parasite within oysters. The increased *P*. *marinus* prevalence in these experiments was seen at DO concentrations that did not increase oyster mortality, but were sometimes associated with reduced growth, particularly in the field. Because the severity and frequency of diel-cycling hypoxia vary spatially within and among estuaries, diel-cycling hypoxia potentially contributes to landscape-level variability in *P*. *marinus* infection dynamics. Although we did not detect effects on oyster mortality during our experiments, the increased prevalence and intensity of infections associated with diel-cycling hypoxia could also result in increased mortality over longer periods of time as infections progress.

Both natural and anthropogenically driven spatial variation in environmental parameters can result in spatial variation in disease at a range of scales through direct effects on the host, the pathogen, or both [46–48]. At large scales (e.g., whole coasts), temperature sets geographic boundaries of *P*. *marinus* epizootics, with northern boundaries limited by cold temperatures and northward spread facilitated both by global warming and interannual variation in temperatures [22]. Within estuaries, salinity can strongly influence landscape-level patterns of host-parasite interactions and resulting epizootics of *P*. *marinus* infections in *C*. *virginica* both through proximate effects and through the influence of salinity on spatial refuges that can affect selection for resistance or tolerance to disease [27,47].

The observed relationships between diel-cycling hypoxia and the epizootology of *P*. *marinus* infections in oysters in our field study were contingent on salinity also being favorable for the transmission and proliferation of *P*. *marinus*. Variability in the prevalence and intensity of *P*. *marinus* infections among our 14 field sites, which were dispersed over a north-south distance of about 150 km, was, however, best explained by the combination of salinity and diel-cycling hypoxia rather than salinity alone. Surveys of oyster disease status consistently demonstrate considerable variability in the prevalence and intensity of *P*. *marinus* among sites in MD waters of Chesapeake Bay with similar salinities (e.g.,[49]). In our study, low oxygen appeared to create greater variation among sites in 2008, the lower salinity year, than during 2009. Thus, environmental factors influencing *P*. *marinus* distributions and dynamics may be hierarchical with temperature setting the broad geographic boundaries, salinity determining within-estuary zones of highest prevalence and intensity, and the frequency and severity of diel-cycling hypoxia especially important in explaining variation in *P*. *marinus* outbreaks at intermediate to local spatial scales—i.e., within salinity zones—and during years with lower than average salinities when salinity alone does not result in near 100% infection prevalence.

Our experiments do not explicitly distinguish between effects of diel-cycling hypoxia on hosts versus effects on the pathogen itself to allow us to definitively identify mechanisms responsible for increased transmission or proliferation of *P*. *marinus*. However, the combination of a likely reduction in hemocyte activity [34], reduced filtration of infective cells, and increased infection rates at similar densities of infective waterborne *P*. *marinus* cells point to diel-cycling hypoxia acting on the host immune response as the most likely mechanism. Previous laboratory research by Boyd and Burnett [34] found that 2-d exposure to low DO reduced production of reactive oxygen intermediates (a key component of oyster defense against pathogens) by stimulated oyster hemocytes. In contrast, reduced feeding during periods of low oxygen that is not completely compensated for during periods of high oxygen should reduce ingestion of *P*. *marinus* cells, and therefore, reduce risk of acquiring infections. Waterborne qPCR assays provided no evidence that low oxygen increased the abundance of *P*. *marinus* supplied to our initially uninfected oysters. Finally, there is no evidence that a low oxygen environment directly increases the virulence of *P*. *marinus*.

Use of RFTM rectal tissue assays to detect *P*. *marinus* allowed us to process large numbers of oysters but likely resulted in miss-classifying some oysters as negative when, in fact, they had extremely light infections [37]. Although the increase in total prevalence caused by exposure to low DO in this study could, therefore, be a combination of new infections and progression of extremely light infections, our results provide an ecologically meaningful measure of the effects of this environmental parameter on disease dynamics.

Our laboratory experiments indicate that diel-cycling hypoxia alone, when frequent, recurring over extended periods of time, and especially when severe, can affect dynamics of *P*. *marinus* infections in eastern oysters. Monitoring conducted 0.3 to 0.5 m above the bottom at shallow, nearshore sites in the Chesapeake Bay and its subestuaries indicated that the frequency and severity of night through early morning hypoxia varies both spatially and among years. Some sites (e.g., the St. Mary’s R. station) experienced DO concentrations below 0.5 mg l^-1^ on most nights during July and August while other sites (e.g. in the Honga R. and the Choptank R. near Mulberry Pt.) remained above 50% saturation. The current monitoring dataset indicates that sites where severe hypoxia (e.g., 0.5 mg l^-1^ DO) occurs at the frequency simulated in our laboratory experiment are not common in Chesapeake Bay. However, monitoring conducted 0.3–0.5 m above the bottom may underestimate the severity of hypoxia within benthic oyster reefs. Dissolved oxygen we measured on 10 mornings during June-August 2014 in 2 m of water in the Rhode River averaged 0.64±0.19 mg l^-1^ higher at 0.7 than at 0.2 m above the bottom (i.e. a difference in 0.5 m depth). Oxygen concentrations experienced by oysters within the reef matrix may also be lower than near-bottom measurements because oxygen demand by oyster reefs is high. For example, oxygen demand on a restored oyster reef in Chesapeake Bay was 20 times that of nearby unrestored bottom [33]. We therefore suspect that a greater percentage of benthic habitat in Chesapeake Bay experiences conditions similar to our most severe laboratory treatments than data from the shallow water monitoring program suggest. In addition, severe, frequent diel-cycling hypoxia occurs in other estuaries and lagoons.

We also we found an increase in *P*. *marinus* infection prevalence and intensity, and decreased growth, associated with less frequent and less severe hypoxia in our field experiments than conditions we tested in the laboratory, suggesting that co-occurring stressors might have added to or exacerbated effects of hypoxia in the field. One potentially important difference between the field and laboratory is that daily minimum pH and daily minimum DO are generally correlated in the field because the consumption of oxygen and release of CO_2_ are both products of respiration (Fig. 1c; see also [50,51]). In contrast, mean daily minimum pH in our laboratory experiments was similar across DO treatments. pH in our low oxygen treatments ranged from only 0.06 less to 0.17 higher than our in our controls, as compared to full pH unit day-night swings that are common in the field (e.g., [11]). Boyd and Burnett [34] found reduced reactive oxygen intermediate production with exposure to low pH. Sensors used at our monitored field sites were calibrated with low-isotonic NBS standards, so comparing sites across a broad range of salinities (including sites in both MD and VA waters) requires some caution in interpreting results. There was a positive correlation between mean daily minimum DO and mean daily minimum pH at the 14 field sites with pH monitoring (Spearman Rank Correlation R = 0.58, P = 0.04). However, statistical models (including year and a possible stressor) that were significant when mean daily minimum DO was included as the stressor (see Results), were not significant when mean daily minimum pH was substituted as the stressor (initially uninfected oysters: overall model for infection prevalence *F*_*2*,*10*_ = 1.41, P = 0.29, pH *F*_*1*,*10*_ = 0.19, P = 0.67; overall model for infection intensity *F*_*2*,*10*_ = 0.38, P = 0.69, pH *F*_*1*,*10*_ = 0.01, P = 0.93). Nevertheless, the combination of previous laboratory results [34] and differences between our lab and field results indicate that more tightly controlled and targeted laboratory experiments are required to resolve the effect of acidification on *P*. *marinus* infection dynamics.

Spatial variation in the density of waterborne *P*. *marinus* could also have influenced our field results. However, that would require spatial variation in local waterborne *P*. *marinus* densities to be strongly influenced by, or at least correlated with, spatial patterns of diel-cycling hypoxia. Such a pattern would support, rather than negate, our interpretation. In the field, the density of waterborne *P*. *marinus* will be a function of a number of factors including the total number of infected oysters, the intensity of infections, oyster mortality (which increases release of *P*. *marinus*), and hydrodynamics that influence transport. Data are not available to estimate these parameters and we were not able to perform waterborne qPCR assays with the temporal resolution and duration needed to evaluate *P*. *marinus* densities throughout the field deployment. Our goal in mixing initially infected and initially uninfected stocks of oysters within cages was to swamp local conditions. Close proximity to infected oysters is considered a major factor contributing to acquisition of new *P*. *marinus* infections by young oysters, and removing older, potentially infected, oysters from restoration sites prior to seeding has become common practice in some areas [52].

Growth rates were also substantially higher in the field than in the laboratory experiments. Phytoplankton available to oysters was undoubtedly higher in the field in spite of the flow-through water supply in the laboratory. Chlorophyll *a* measured in the inflow to aquaria was substantially lower than that measured near the lab’s seawater intake in the Rhode R., and oysters depleted phytoplankton in the tanks. If nutritional state contributed to susceptibility to *P*. *marinus* infections and exacerbated effects of low oxygen, then effects of low oxygen exposure should have been more marked in laboratory experiments than in the field. This was not the case. Adequate (or surfeit) nutrition can mask effects of stressors by providing the means to compensate for energetic demands of suboptimal conditions [53]. In our case, higher growth rates of field oysters may have, instead, facilitated detection of diel-cycling hypoxia effects on growth, and did not negate effects of stressors on acquisition and progression of infections. As with infection acquisition and progression, the correlation between pH and dissolved oxygen may have contributed to growth effects detected in field experiments. Acidification has been shown to reduce post-settlement bivalve growth both alone (e.g. [54]) and in combination with hypoxia [55].

Analyses of wet weights in 2010 laboratory experiment, and shell heights in field experiments indicated that exposure to diel-cycling hypoxia does negatively affect growth rates of 1yo oysters. IVF measurements in experimental aquaria indicated that filtration rates of 1yo and older oysters were reduced during exposure hypoxia, although at least older oysters increased filtration rates in a compensatory manner as oxygen concentrations cycled back towards normoxia. Growth rates at the site with the lowest mean daily minimum oxygen concentration, and highest percentage of days with oxygen concentrations below 2 mg l^-1^ (Site 10: St. Mary’s R.) were less than half that at sites where oxygen concentrations remained high at night. Detection of cycling DO effects on growth rates of older oysters were likely hindered by the general low growth rates and confounded by reproduction, which was not measured in either field or laboratory experiments.

## Conclusions

Diel-cycling hypoxia is caused by a combination of natural factors including biomass production, restricted water exchange, and weather conditions that reduce photosynthesis and reaeration, and is exacerbated by anthropogenic nutrient loads that stimulate biomass and production with a consequent increase in respiration that consumes dissolved O_2_ [9,10]. Our results suggest that by worsening diel-cycling hypoxia, anthropogenic nutrient loadings can increase oyster disease problems in shallow waters. In addition, spatial variation in the severity or frequency of diel-cycling hypoxia can contribute to landscape-level spatial variability in disease in estuaries with heterogeneous nutrient loads, physics and salinity.

Oysters were historically abundant in shallow waters in Chesapeake Bay. Although overfishing was the major cause of their decline [23,56], disease mortality has contributed in recent decades. Hypoxia, including diel-cycling hypoxia may influence the success of oyster restoration via direct effects and by influencing disease dynamics, and should be considered in siting of restoration programs. The presence of diel-cycling hypoxia in shallow water should not necessarily preclude use of sites for oyster restoration, however. Unlike deep channel areas, the entire shallow nearshore water column is likely accessible to oysters during the daytime, high oxygen phase of the diel cycle. These sites may, therefore, be the ones where oyster restoration done at sufficiently large scales could tip the balance from conditions in which water quality limits the ecosystem services provided by oysters to one in which the full ecological benefits of oyster restoration can be realized.

Although our experiments focused on oysters and Chesapeake Bay, it is important to consider that the issues of diel-cycling hypoxia and aquatic disease (including, but not limited to other forms of Perkinsosis) are prevalent worldwide. Diel-cycling hypoxia occurs in many shallow estuaries, embayments, and coastal lagoons [57,58]. Furthermore, short-term exposure to hypoxia has been shown to decrease immune responses of a variety of marine invertebrates [59–64]. The potential for temporally varying conditions that increase the local prevalence or intensity of infectious diseases may be an underappreciated cause of spatial variation in disease dynamics in natural systems. Our study extends the understanding of how the physical environment can modulate disease processes in aquatic environments by linking high-frequency variability in key environmental parameters (DO in the present case) to spatial patterns of disease occurrence and severity at local-to-intermediate spatial scales.

## Supporting Information

S1 Fig Dissolved oxygen concentrations at field experiment sites vs Julian date. All field experiment sites are shown except for the Choptank R. at Mulberry Point (Site 1) and the St. Mary’s R. (Site 10), which are illustrated in Fig. 1 of the main paper. Site numbers correspond to numbering in Fig. 2 in the main paper and S1 Table.
(PDF)

S2 Fig Temperature and salinity measured within 0.5 m above the bottom at field experiment sites. (PDF)

S3 Fig Prevalence and intensity of *P*. *marinus* infections in 2 and 3 yo oysters in laboratory experiments (mean ± SE). Only infected individuals (i.e., Mackin score of 0.5 or greater) were included in intensity calculations.
(PDF)

S1 Methods Additional field experiment methods: (1) Acclimation and preparation for deployments; and (2) Analyses. (PDF)

S2 Methods Additional methods for laboratory experiments: (1) Acclimation and preparation for experiments; (2) Statistical analysis note; and (3) qPCR methods to test for density of waterborne *Perkinsus marinus* cells in laboratory experiments. (PDF)

S1 Results Additional results for laboratory experiments: (1) 2009 experiments; (2) 2010 experiment. (PDF)

S1 Table Locations of experimental oyster deployments in 2008–09. Numbers correspond to the site code in Fig. 1 in the main manuscript and S1 Fig.
(PDF)
