# Supplementary figures and images for: Landscape-Level Variation in Disease Susceptibility Related to Shallow-Water Hypoxia
Source: PLoS One. 2015 Feb 11;10(2):e0116223. doi: 10.1371/journal.pone.0116223 (PMC4324988; doi:10.1371/journal.pone.0116223)

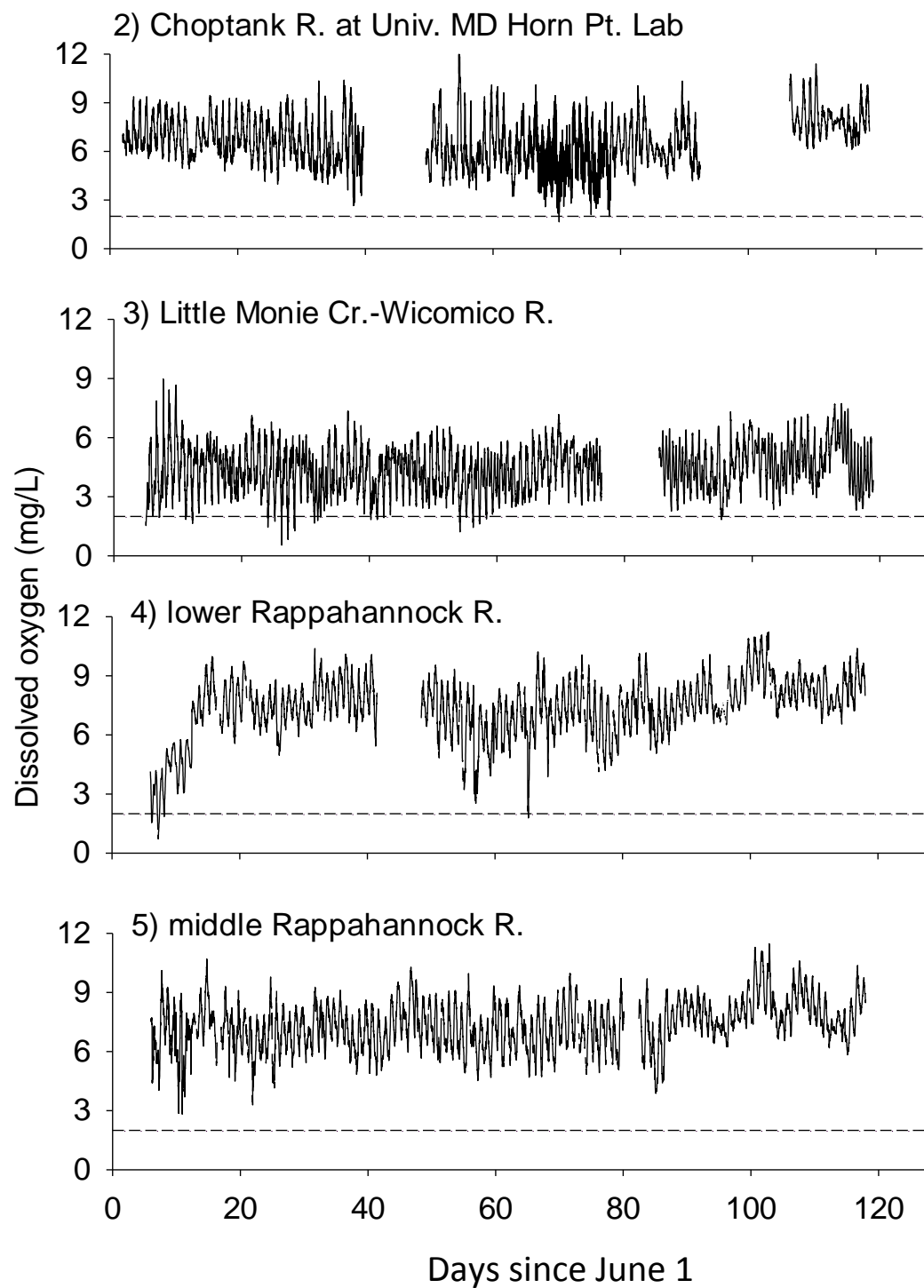

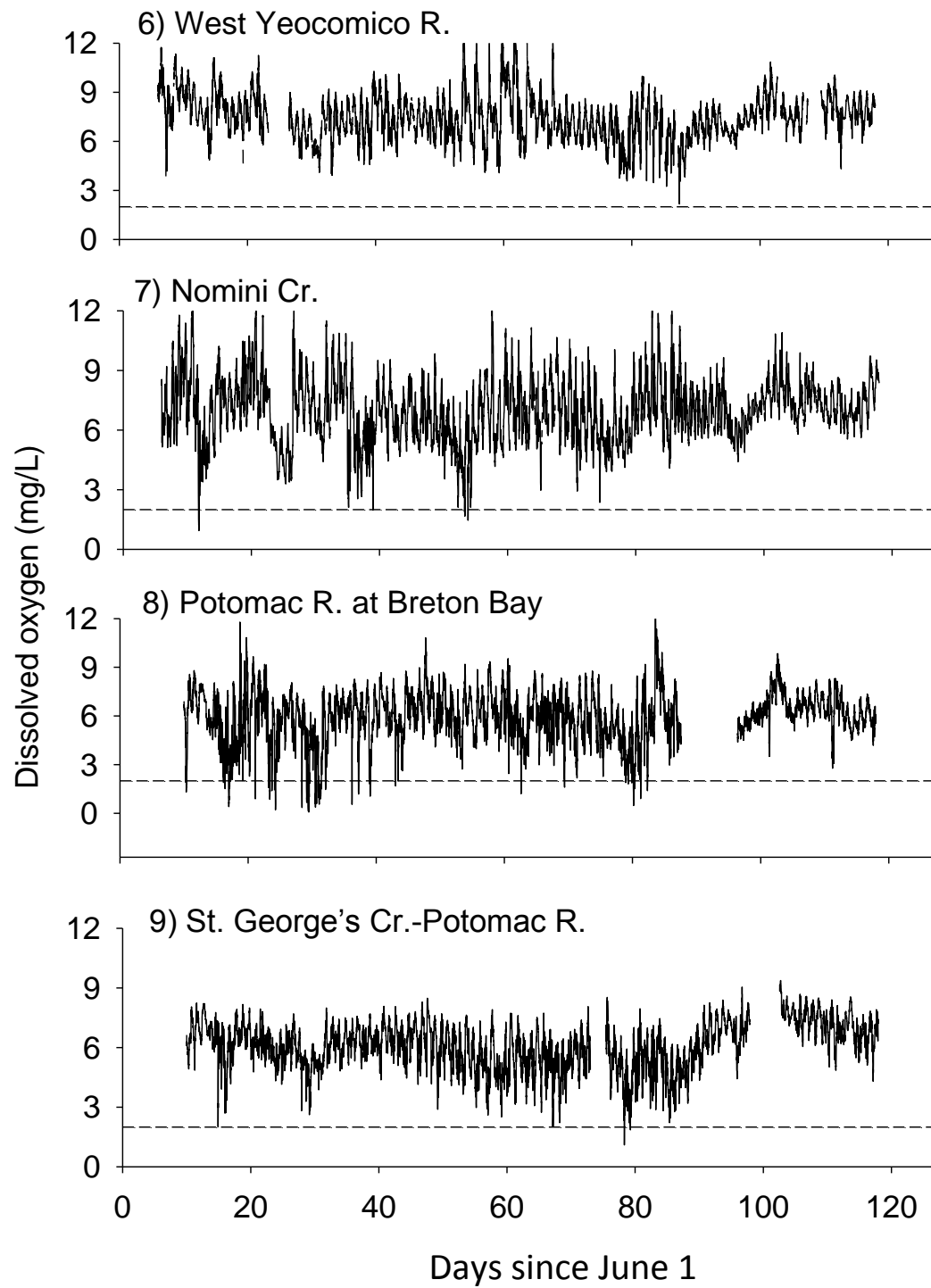

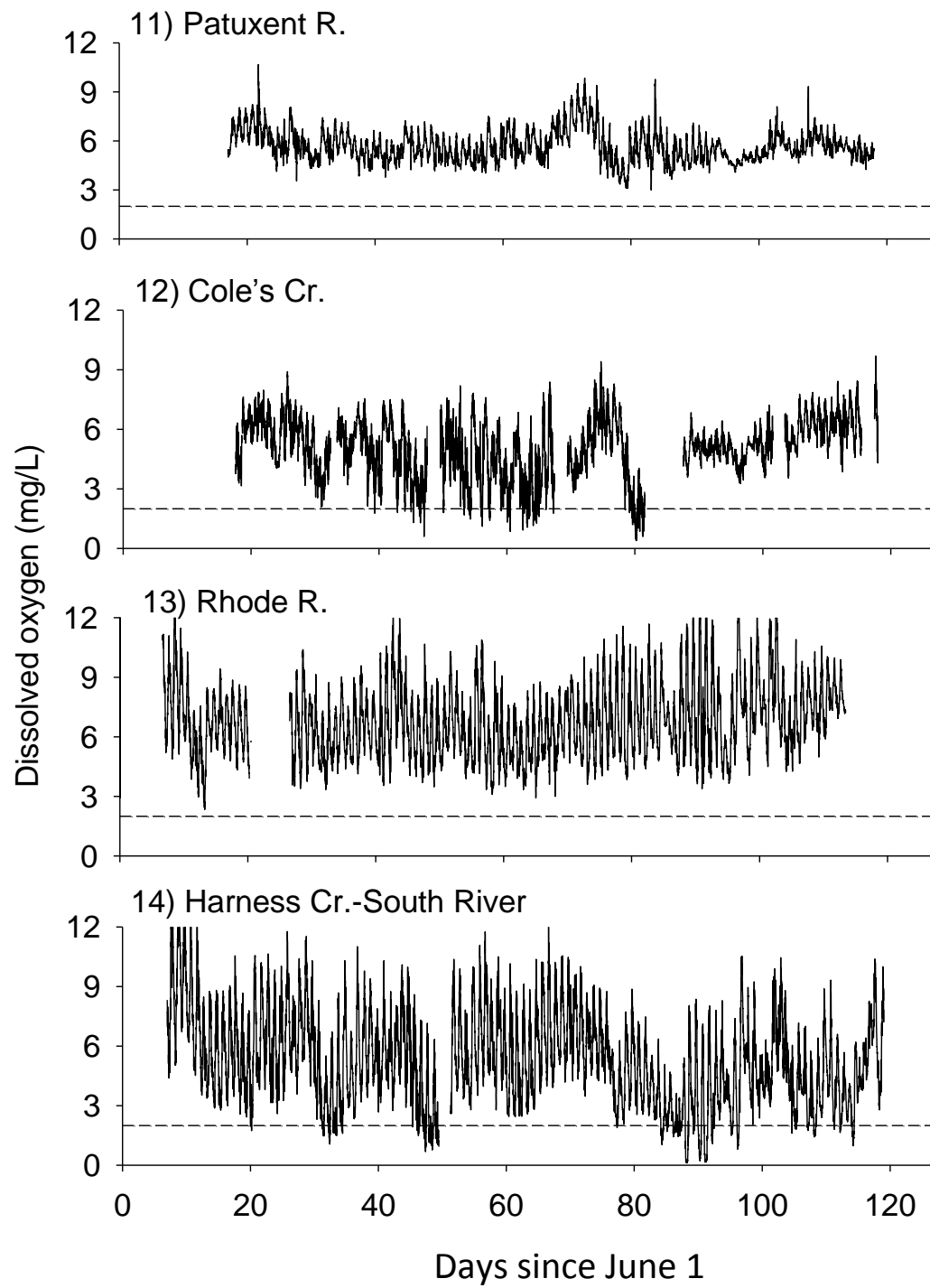

Supplement: S1 Fig — All field experiment sites are shown except for the Choptank R. at Mulberry Point (Site 1) and the St. Mary’s R. (Site 10), which are illustrated in Fig. 1 of the main paper. Site numbers correspond to numbering in Fig. 2 in the main paper and S1 Table. (PDF) [file pone.0116223.s001.pdf]

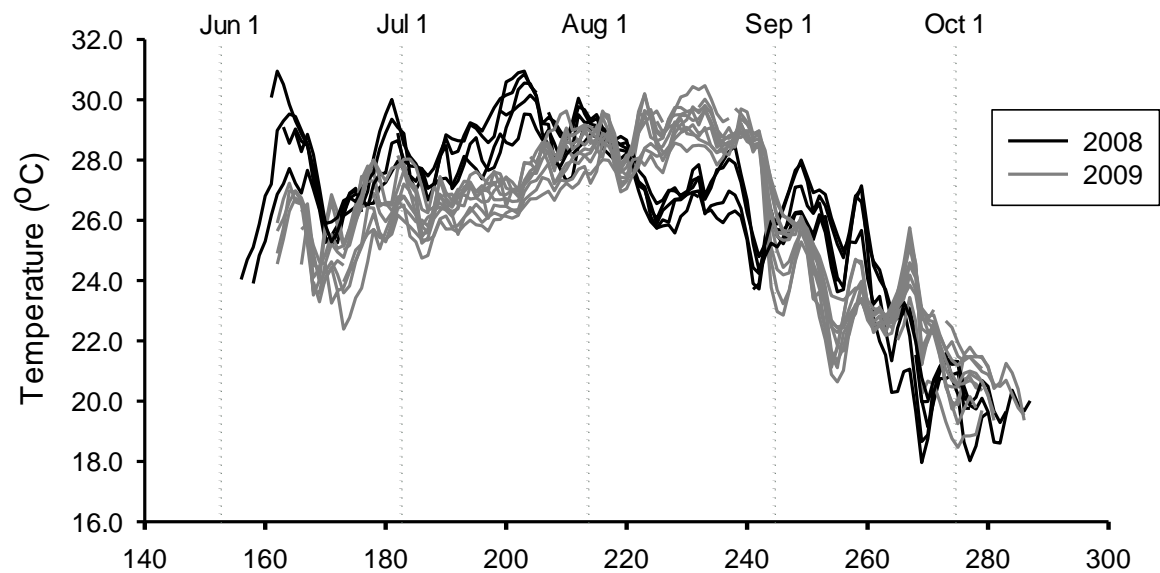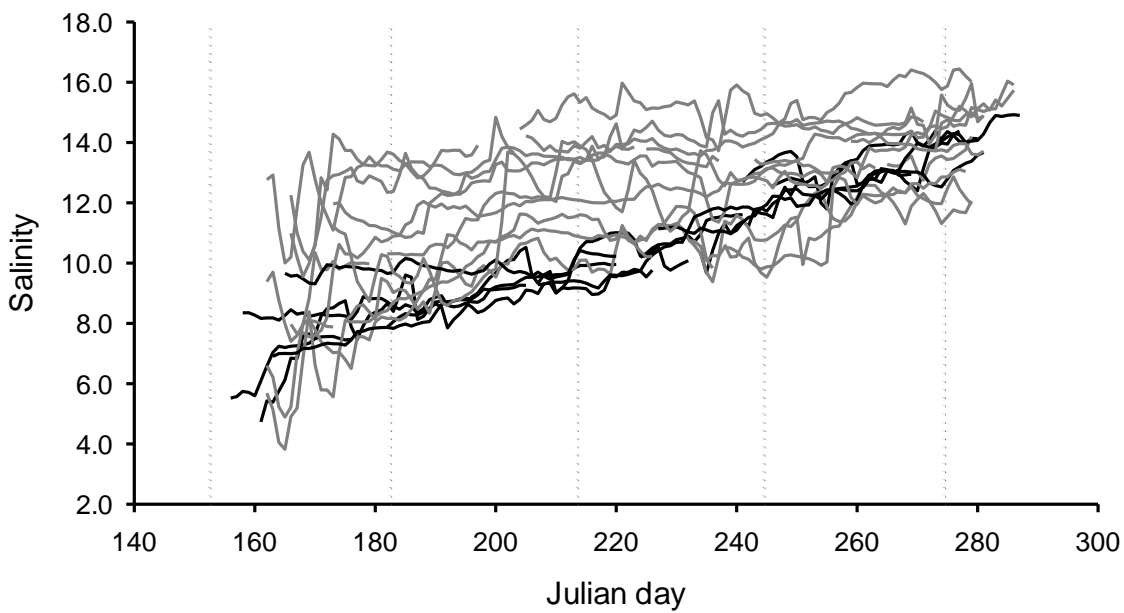

Supplement: S2 Fig — (PDF) [file pone.0116223.s002.pdf]

2009

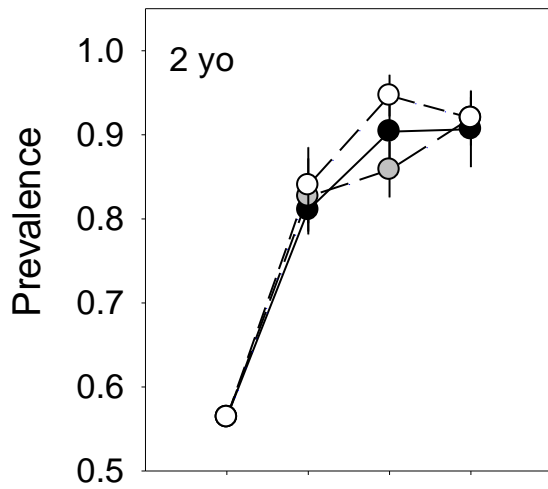

2010

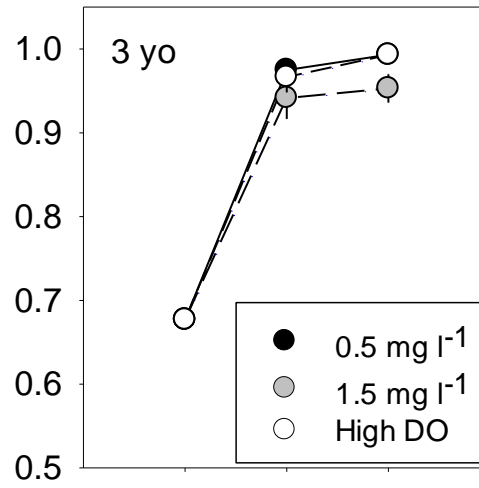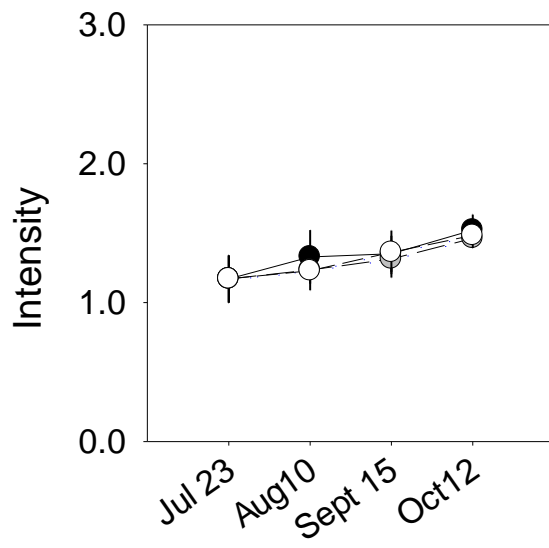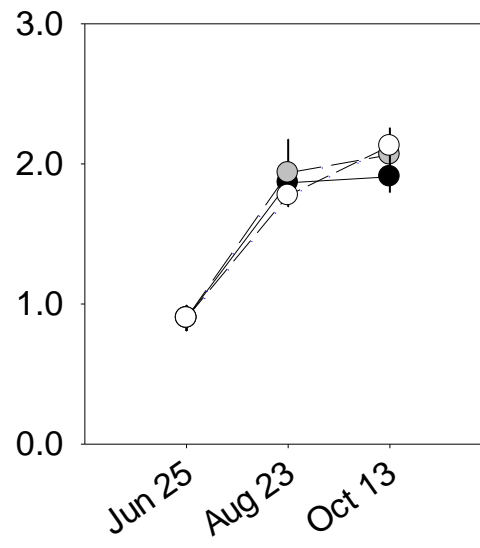

Supplement: S3 Fig — Only infected individuals (i.e., Mackin score of 0.5 or greater) were included in intensity calculations. (PDF) [file pone.0116223.s003.pdf]
