# Supplementary material for: Landscape-Level Variation in Disease Susceptibility Related to Shallow-Water Hypoxia
Source: PLoS One. 2015 Feb 11;10(2):e0116223. doi: 10.1371/journal.pone.0116223 (PMC4324988; doi:10.1371/journal.pone.0116223)
Supplement: S1 Methods — (PDF) [file pone.0116223.s004.pdf]

Methods S1.

Additional Field Experiment Methods

Acclimation and preparation for deployments

Prior to deployment, initially-uninfected and infected hatchery oysters were held in tanks at the Smithsonian Environmental Research Center (SERC; Edgewater, MD). They were supplied with Rhode R. water, which was treated with ultraviolet sterilization before being discharged. Two days before deployment, oysters' shells were marked with nail-polish to identify oysters as belonging to the initially-uninfected or initially-infected stock. Numbered tags were glued to the right valve of 48 randomly-selected individuals of each group destined for each deployment site in order assess individual oyster growth. Shell heights were measured to the nearest mm with a flexible plastic ruler following methods used by MD-DNR. Shells of dead oysters ("boxes") were recorded and removed when cages were opened for cleaning.

Analyses

If exposure to low DO increases oyster susceptibility to infection by *P. marinus*, disease prevalence, intensity, and perhaps mortality in experimental oyster deployments were expected to be greatest at test sites at which diel-cycling hypoxia was most frequent and/or severe, whereas oyster growth was expected to be highest at sites where diel-cycling hypoxia was least frequent and/or severe. Modeling approaches used to evaluate the preceding hypotheses included salinity, which is known to influence the transmission and progression of *P. marinus*

infections, and was periodically favorable for disease transmission at all sites—even at sites with average salinity less than the critical threshold.
