# Supplementary material for: Landscape-Level Variation in Disease Susceptibility Related to Shallow-Water Hypoxia
Source: PLoS One. 2015 Feb 11;10(2):e0116223. doi: 10.1371/journal.pone.0116223 (PMC4324988; doi:10.1371/journal.pone.0116223)
Supplement: S2 Methods — (PDF) [file pone.0116223.s005.pdf]

Methods S2. Additional methods for laboratory experiments.

Acclimation and preparation for experiments

Oysters were held at SERC in flow-through raw Rhode R water until being placed in experimental aquaria. Oysters were acclimated to aquaria and a reversed light-dark cycle for 6 days before the start of DO treatments. Reversed light-dark cycles allowed technicians to monitor oxygen, IVF, and other conditions during normal working hours. DO, temperature, pH and salinity were measured at least twice daily on days that DO was manipulated (prior to ramping down DO and at the end of the target low-DO phase of the cycle) and once daily when DO was not manipulated. Oysters were washed with Rhode R. water and tanks were cleaned approximately every 2 weeks to reduce the build-up of feces and sediment in tanks and to remove invertebrates that entered through the sea water system. All effluent was treated with UV sterilization before release. Three-year old oysters used in 2010 experiments were from the same cohort as the 2yo oysters used in 2009.

We noted mortality, but did not remove dead individuals with tissue present because these were a potentially important source of infection transmission. Presumably because of differences in consumer abundance and diversity in the laboratory aquaria and field cages, and the frequency of maintenance, dead oysters often still contained tissue when discovered in our laboratory experiments, but all that remained in the field were empty shells.

Statistical analysis note

The block effect (room position) was only included in final statistical analyses where it improved our ability to detect effects of DO treatment.

qPCR Methods to Test Initial Disease Status in 2009 Laboratory Experiments

In 2009, we evaluated the severity of *P. marinus* infections in a subset of oysters using qPCR, along with RFTM assays in order to provide a more sensitive measure of infection prevalence and a more quantitative measure of disease intensity. The methodology employed followed the PMAR assay described in Gauthier et al. (2006) with the following exceptions: DNA from each individual was extracted from 10mg (wet weight, blotted) of gill and mantle tissue using the Qiagen *DNeasy Blood & Tissue Kit*; 7 standards were created by spiking 10mg of gill and mantle tissue from uninfected oysters (acquired from Mook Sea Farm, 121 State Route 129, Walpole ME, 04573) with 5×10^0, 5×10^1, 5×10^2, 5×10^3, 5×10^4, 5×10^5, and 5×10^6 monovalent, mono-dispersed *P. marinus* cells (isolate ATCC 50439; *P. marinus* cells provided by C. Dungan, MD Dept. of Natural Resources); per-well volumes of Taqman Universal Master Mix II (2X), 25X primer/probe mix, DNase-free water, and DNA sample (40ng/µl) were 12.5, 1, 6.5, and 5 µl, respectively, for a total reaction volume of 25µl; qPCR was completed using a MJ Research, Inc. (now BioRad) Opticon 2 Real Time PCR System. The statistical relationship between density of *P. marinus* in standards and threshold cycle (C_T) from the qPCR analysis was highly significant (Appendix B, Figure 1).

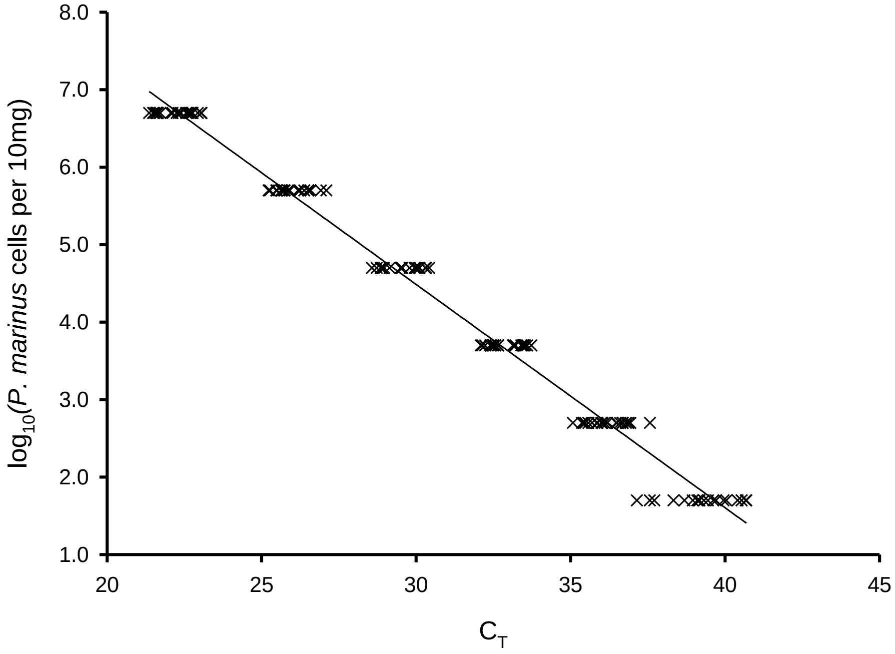

Appendix B, Figure 1. Relationship between *P. marinus* cell density and threshold cycle (C_T) from qPCR analysis of standard samples. Regression line: $\log_{10}$(*P. marinus* cells per 10 mg) = 13.13 – 0.29*C_T; $r^2 = 0.99$, $P < 0.01$.

qPCR Methods to Test for Density of Waterborne Perkinsus marinus Cells in Laboratory Experiments

Water samples were collected from individual aquaria on 8 dates and from water holding tanks used to supply water to aquaria, filtered on onto a 47 mm diameter, 3 µm-pore size Nuclepore filter (Costar, Whatman, Clifton, New Jersey) using a disposable apparatus (Nalgene Nunc International, Rochester, New York) to minimize sample to sample contamination, and stored in 180 µl lysis buffer with 20 µl (100 mg/ml) Proteinase K (Qiagen) until processing at Virginia Institute of Marine Science (VIMS) in Gloucester, Virginia, USA. At VIMS an additional 20 µl

of Proteinase K was added to the filters before incubation overnight at 55°C to lyse the cells for subsequent DNA extraction using the Qiamp stool mini kit (Qiagen). Total DNA recovery was increased by eluting DNA from the column with three buffer (AE) loadings of 100 µl each and mixing the three eluates prior to quantification (Audemard et al., 2004). The standard DNAs used to evaluate the abundance of *P. marinus* in experiment water samples were obtained by spiking a known number of cultured *P. marinus* cells into artificial seawater to obtain a final volume of 250 ml, extracting DNA from the cells collected on a filter, and serially diluting tenfold using the elution buffer. Quantification was performed by qPCR using the *P. marinus* specific primers and probe designed by Gauthier et al. (2006). Reagents concentrations were the following: bovine serum albumin (BSA) at 0.2µg/µl final concentration, 1X TaqMan® Fast Universal Master Mix (Applied Biosystems, Carlsbad, CA), primers and probe were 0.4µM and 0.1µM, respectively, and 1 µl of template in a 10 µl reaction volume. qPCR reactions were run on a 7500 Fast Real-Time PCR machine (Applied Biosystems, Carlsbad, CA) using the fast conditions (95˚C/20s; 45 cycles of 95˚C/3s followed by 60˚C/30s).
