# Supplementary material for: Landscape-Level Variation in Disease Susceptibility Related to Shallow-Water Hypoxia
Source: PLoS One. 2015 Feb 11;10(2):e0116223. doi: 10.1371/journal.pone.0116223 (PMC4324988; doi:10.1371/journal.pone.0116223)
Supplement: S1 Results — (PDF) [file pone.0116223.s006.pdf]

Results S1. Additional results for laboratory experiments.

2009 Experiments

DO concentrations ≤0.5 mg/L above target levels were reached during 47 to 51 days (for the various oyster age and DO treatments) of the 54 days that we manipulated oxygen concentrations. There were no significant differences among starting sizes of oysters (1-way ANOVA for each size class; all p≥0.35). Across all DO treatments, the initial total prevalence and intensity of *P. marinus* infections averaged 3% and 0.5 (n=35), respectively in 1yo oysters and 44% and 1.2±0.2 (n=73) respectively in 2yo oysters that served as the source of infection.

Diel-cycling DO did not affect total prevalence, MHprevalence, or intensity of *P. marinus* infections in 2yo oysters that started the experiment with a high total prevalence of infections (Table 4, Fig. S3). Total prevalence averaged 91 – 92%, MHprevalence averaged 31 – 33%, and intensity averaged 1.9 – 2.1, at the end of the experiment in DO treatments with 2yo oysters. There were no differences in mortality among DO treatments for either age class (Table 4). Mortality ranged from 8.7% in the 1.5 mg/L treatment to 12.4% in the high oxygen controls for 2yo oysters (Table 3). There was also no effect of DO treatment on ending oyster shell height (Table 4).

2010 Experiment

Daily minimum DO concentrations averaged 0.57±0.01 (n=641) in the 0.5 mg /L treatment, 1.50±0.02 (n=641) in the 1.5 mg /L treatment, and 7.08±0.02 (n=641) in controls across both tank sizes on days in which we manipulated tank oxygen concentrations. Daily minimum DO concentrations ≤0.5 mg/L above target levels were reached during 64 days. There were no

significant differences among starting shell heights of oysters ($P \geq 0.1$ for all oxygen treatments and age classes). Initial intensity and total prevalence of *P. marinus* infections in oysters was similar in 2010 as in 2009. Initial total prevalence and intensity of *P. marinus* infections averaged 4% (n=94) and 0.5 ± 0.0 (n=4), respectively in 1yo oysters and 68% (n=99) and 1.3±0.8 (n=67) respectively in 3yo oysters that served as the source of infection. We used a larger sample size than in 2009 in order to increase the likelihood of detecting infected individuals in spite of the low-total infection prevalence in 1yo oysters.

Total prevalence in 3yo oysters at the end of the experiment ranged from 95 ±1.7% in the 1.5 mg/L treatment to 99.3±0.7% in both the 0.5 mg/L and control treatments and although the DO treatment effect was statistically significant (Table 4; LSmeans P=0.025 for both 1.5 mg/L versus controls and 1.5 mg/L versus 0.5 mg/L) the differences among treatments were quite small (i.e. 4% for both comparisons) (Fig. S3). Detection of treatment effects was constrained by the near 100% total infection prevalence in these oysters. Mean intensity in infected oysters ranged from 1.9±0.1 in the 0.5 mg/L treatment to 2.2±0.1 in controls and did not vary significantly among treatments Table 4). Similarly, MHprevalence ranged from 54.7±5.0% in the 0.5 mg/L DO treatment to 58.0±5.0 in the high DO controls, and did not vary among treatments (Table 4).

DO treatment did not affect final shell height or wet weight of 3yo oysters (Table 4). Mortality of 3yo oysters varied significantly among DO treatments (Table 4), but was not increased by exposure to hypoxia. Planned comparisons indicated that 3yo oysters in controls had significantly higher mortality than in the 0.5 mg l^{-1} DO treatment (P=0.016).
