# Supplementary material for: Landscape-Level Variation in Disease Susceptibility Related to Shallow-Water Hypoxia
Source: PLoS One. 2015 Feb 11;10(2):e0116223. doi: 10.1371/journal.pone.0116223 (PMC4324988; doi:10.1371/journal.pone.0116223)
Supplement: S1 Table — Numbers correspond to the site code in Fig. 1 in the main manuscript and S1 Fig. (PDF) [file pone.0116223.s007.pdf]

Table S1.

Locations of experimental oyster deployments in 2008-09. Numbers correspond to the site code in Figure 1 in the main manuscript and Fig. S1.

Site Number	Year Sampled	Location description
1	2008	Choptank R. at Mulberry Pt. (38.7494º N, -76.2440º W)
2	2008	Choptank R. at Univ. MD Horn Pt. Lab. (38.5935º N, -76.1288º W)
3	2008	Little Monie Cr.-Wicomico R. (38.2086º N, -75.8046º W)
4	2009	lower Rappahannock R. (37.6209º N, -76.5467º W)
5	2009	middle Rappahannock R. (37.8220º N, -76.7503º W)
6	2009	West Yeocomico R. (38.0290º N, -76.5519º W)
7	2009	Nomini Cr. (38.1319º N, -76.7176º W)
8	2009	Potomac R. at Breton Bay (38.2590º N, -76.6714º W)
9	2009	St. George’s Cr.-Potomac R. (38.1310º N, -76.49330º W)
10	2009	St. Mary’s R. (38.1894º N, -76.4338º W)
11	2009	Patuxent R. (38.3175º N, -76.4515º W)
12	2009	Cole’s Cr. (38.3392º N, -76.4334º W)
13	2008	Rhode R. (38.8860º N, -76.5415º W)

14	2008	Harness Cr.-South River (38.9365º N, -76.5074º W)
